# Tuning dynamic DNA- and peptide-driven self-assembly in DNA–peptide conjugates†

**DOI:** 10.1039/d2sc02482a

**Published:** 2022-11-30

**Authors:** Emerald R. Taylor, Akiko Sato, Isobel Jones, Prashant G. Gudeangadi, David M. Beal, James A. Hopper, Wei-Feng Xue, Michael R. Reithofer, Christopher J. Serpell

**Affiliations:** School of Chemistry and Forensic Science, University of Kent Ingram Building Canterbury Kent CT2 7NH UK c.j.serpell@kent.ac.uk; School of Biosciences, University of Kent Stacey Building Canterbury Kent CT2 7NJ UK; Department of Inorganic Chemistry, University of Vienna Währinger Straße. 42 1090 Vienna Austria michael.reithofer@univie.ac.at

## Abstract

DNA–peptide conjugates offer an opportunity to marry the benefits of both biomolecular classes, combining the high level of programmability found with DNA, with the chemical diversity of peptides. These hybrid systems offer potential in fields such as therapeutics, nanotechnology, and robotics. Using the first DNA–β-turn peptide conjugate, we present three studies investigating the self-assembly of DNA–peptide conjugates over a period of 28 days. Time-course studies, such as these have not been previously conducted for DNA–peptide conjugates, although they are common in pure peptide assembly, for example in amyloid research. By using aging studies to assess the structures produced, we gain insights into the dynamic nature of these systems. The first study explores the influence varying amounts of DNA–peptide conjugates have on the self-assembly of our parent peptide. Study 2 explores how DNA and peptide can work together to change the structures observed during aging. Study 3 investigates the presence of orthogonality within our system by switching the DNA and peptide control on and off independently. These results show that two orthogonal self-assemblies can be combined and operated independently or in tandem within a single macromolecule, with both spatial and temporal effects upon the resultant nanostructures.

## Abstract 2

DNA and peptide nanotechnologies can be interfaced to create hierarchical and emergent superstructures, which evolve with time.

## Graphical abstract

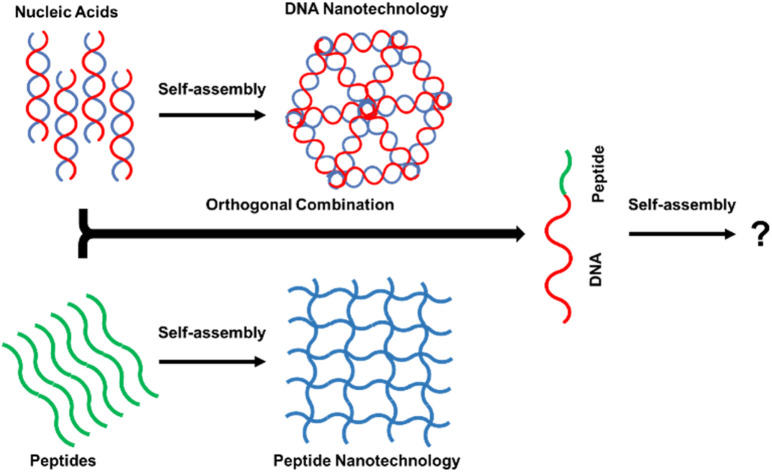

## Introduction

Biomolecular chemistry opens up a wide array of applications in the confluence of biology and chemistry.^1^ DNA and peptides in particular offer contrasting and complementary properties in their supramolecular chemistry. DNA has highly site-specific recognition driven by Watson–Crick base pairing and the formation of the double helix; this can be manipulated to achieve more complex structures, such as those explored in DNA nanotechnology.^2^ Peptides, with a large library of monomers, have a high level of variation and self-assembly motifs, giving more diversity and fluidity of structure.^3–6^ DNA–peptide conjugates therefore exploit the properties of each of their components, but also overcome their individual weaknesses. For example, peptides can benefit from the rigid control of self-assembly that DNA nanotechnology provides, while DNA can be enhanced by the increased biological and mechanical stability which peptides supply.^1,7^ This makes DNA–peptide conjugates ideal candidates for exploration in medicinal and therapeutic fields,^6,8–10^ as well as nanoscale control and assembly of bioactive materials and catalysts.^11^

DNA–peptide conjugate systems have recently been rising in prominence. Buchberger *et al.* have explored nanoscale assembly of DNA origami blocks connected *via* coiled-coil peptides to yield controllable nanostructures. By varying the number of handles, the team controlled assembly to yield, dimers, trimers and nanofibers.^1^ Diphenylalanine nucleic acid conjugates have been shown to self-assemble into nanoparticles.^12^ The structural control of these systems has been explored through external stimuli such as salt, pH, concentration and heat.^2,13,14^ Self-assembly has been driven by either of the DNA or peptide components, or jointly.^15–17^ In general, a hierarchical approach has been used in which pre-assembled DNA or peptide units are subsequently interlinked *via* the other component.^7,18^ The aging of self-assembled structures has not been explored in the DNA–peptide conjugate field. However, it is well established in amyloid peptide structural studies.^19^ Studying the way that amyloid proteins and peptides self-assemble over time has greatly increased our insight into amyloid fibres.^20–22^ These same insights could be beneficial to the study and understanding of DNA–peptide self-assembly, as well as allowing the exploration of the dynamic properties that could be present within these systems.^23–25^

We here report the first conjugates of DNA and β-turn-forming peptides, and show how their self-assembly in space and time is influenced by each of the components separately and together.

## Results and discussion

Our system is comprised of two complementary DNA sequences modified with a bifunctional crosslinker and attached by copper-free click reaction to the β-turn peptide, N_3_–ILVAGK–NH_2_. ILVAGK is a hexapeptide with a hydrophobic block proceeded by increasingly hydrophilic residues.^26,27^ This gives ILVAGK its amphiphilic character which promotes a progressive self-assembly from random coil to α-helical and finally β-turn conformations.^27^ This peptide is pH- and salt-sensitive, and known to self-assemble to form hydrogel systems, with potential use in medicine.^28^

**Fig. 1:**
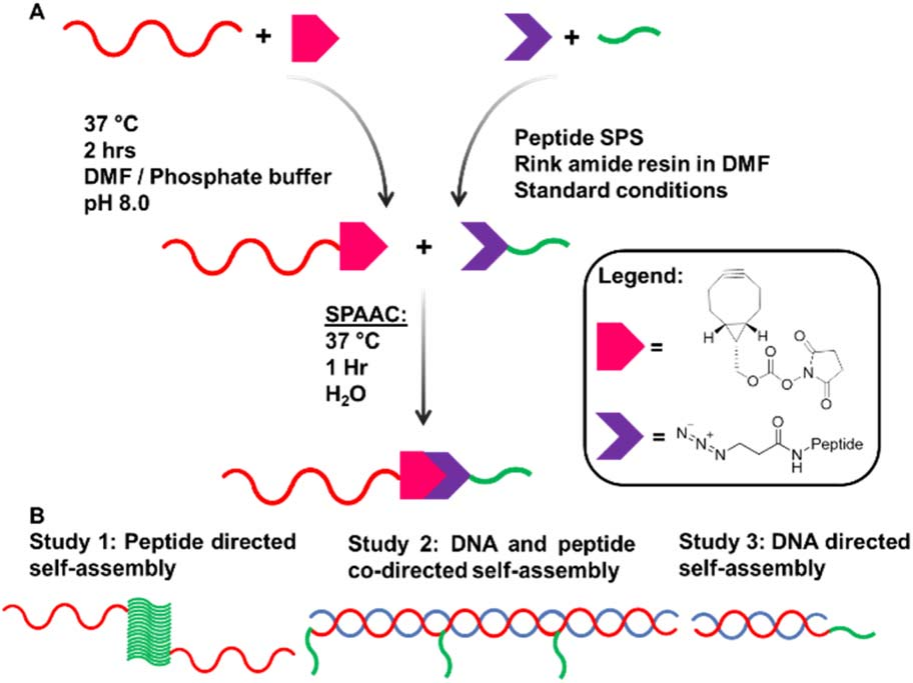
(A) Synthesis pathway to achieve DNA–peptide conjugates by solution phase modification. (B) Schematic representation of studies 1, 2 and 3.

Complementary 15mer oligonucleotide sequences A and A′ were used (see ESI† for sequences). Oligonucleotides with a 5′ T_5_ spacer, followed by C6 primary amine termination (A–NH_2_ and A′–NH_2_), were used for attachment of peptides through modification with bicyclononyne *via* an activated *N*-hydroxysuccinimide (BCN–NHS) to achieve a carbamate linkage.^29^BCN–NHS was dissolved in dimethylformamide (DMF) and added to oligonucleotides A–NH_2_ and A′–NH_2_ in phosphate buffer, before being heated to 37 °C for 2 hours. Crude BCN–oligonucleotides were purified by size exclusion chromatography before DNA–peptide conjugation. A–BCN and A′–BCN were then conjugated to N_3_–ILVAGK–NH_2_ through strain promoted alkyne–azide click reaction (SPAAC) (Fig. 1a).^30^ Oligonucleotide–peptide conjugates were purified by anion exchange and size exclusion chromatography before a successful conjugation was confirmed by polyacrylamide gel electrophoresis (PAGE) and mass spectrometry. Masses were observed for DNA–peptide conjugates at 7150.61 [M − H]^−^ and 7128.63 *m*/*z* [M − H]^−^ for A–ILVAGK and A′–ILVAGK, respectively (see ESI Fig. S4 and S5†).

Three studies were conducted to investigate how the self-assembly of this system changes when driven by peptide and/or DNA self-assembly (Fig. 1b). Study 1 explored the way in which the presence of DNA–peptide conjugates affected the self-assembly of the parent peptide, ILVAGK. DNA hybridisation was added as a second driving force in Study 2, modulating and linking prior substructures. Finally, Study 3 investigated whether the two self-assembling systems were orthogonal by switching each type of assembly on and off. The studies were monitored over a period of 28 days to observe the dynamic nature of this system and investigate the stability of the self-assemblies produced, using atomic force microscopy (AFM), dynamic light scattering (DLS) and circular dichroism (CD). A variety of nanostructures were observed, which we have classified as the following morphologies; small and large flakes, fibre-like, fractal-like, dot-like and network-like (Fig. S1†).^31^

**Fig. 2:**
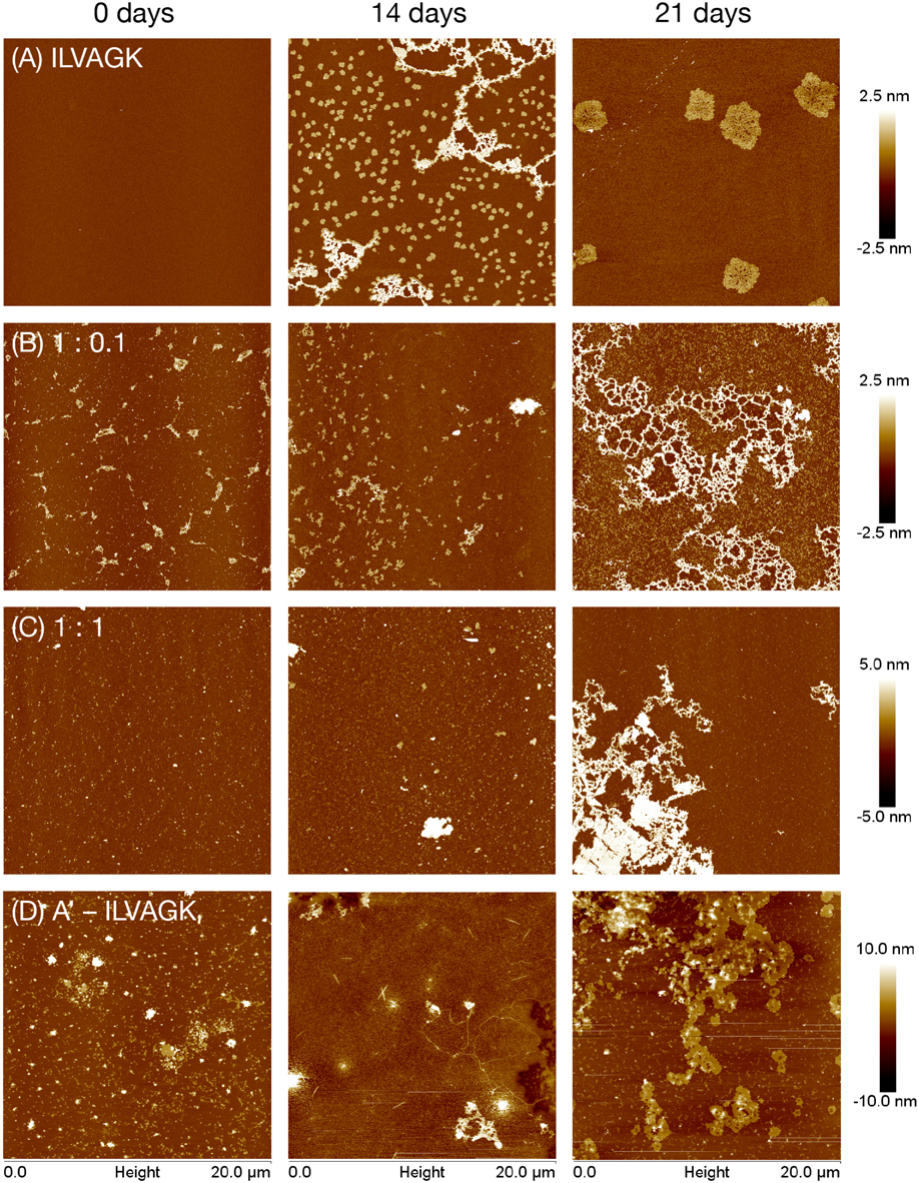
AFM images showing change in self-assembly of ILVAGK and A′–ILVAGK DNA–peptide conjugate in varying ratios: (A) pure ILVAGK; (B) 1 : 0.1 ILVAGK : A′–ILVAGK; (C) 1 : 1 ILVAGK : A′–ILVAGK; and (D) pure A′–ILVAGK, after aging in TAMg buffer (see ESI Fig. S8–S13† for full 28 day time course). All AFM samples were deposited at a total peptide concentration of 10 μM.

Study 1 was designed to investigate the effect of conjugation to DNA has upon peptide-driven self-assembly. Tris-magnesium acetate (TAMg) buffer was chosen because its magnesium ions limit electrostatic repulsions, and so will also allow DNA hybridisation to occur later on, in Studies 2 and 3. Samples of 20 μM ILVAGK were doped with varying concentrations of A′–ILVAGK (1, 0.1, 0.01 and 0.001 equiv.) ensuring an overall concentration of 10 μM of [ILVAGK + A′–ILVAGK], and a sample of pure A′–ILVAGK was taken as a comparison. Samples were aged at room temperature over a period of 28 days, with day 0 being the day the samples were prepared. DLS (Fig. S6†) showed little change over the aging period in gross hydrodynamic diameter, with the exception of the pure A′–ILVAGK conjugate which also had a small diameter (∼1 nm peak) which disappeared over the timecourse to be replaced by a large population centred at 1 μm, as with the other samples. This is at the upper end of the instrument's sensitivity and should be taken to include larger structures. There was evolution of the CD spectra in the 1 : 1 and 1 : 0.5 peptide : peptide–DNA samples indicating an internal structural shift (Fig. S7†). In these cases, in contrast to spectra typical of DNA single strands which have a maximum around 220 nm, at the 0 days mark there was a minimum at that wavelength. However, this signal disappeared over the course of the experiment to be replaced by a more classical ssDNA CD spectrum (positive around 220 nm, with a further maximum around 275 nm). This indicates slow relaxation of the DNA from an unstable structure to something closer to its thermal minimum. The same process may have occurred in the more DNA–dilute samples, but is harder to discern due to the weaker signal. The positive CD peak at ∼197 nm is consistent with the β-turn structure and was consistently present. In contrast, the pure DNA–peptide conjugate sample showed the expected ssDNA spectral shape, with little deviation over the timecourse. AFM revealed that the structural changes over the time period were reflected in nanoscale morphology: self-assembly of pure ILVAGK varied over the 28 days (Fig. 2a, S13†). At day 0 no structures were visible on the surface; this changed at 14 days of aging to network-like features with a height between 2 and 3 nm, with small flake structures with an average height and diameter of 1.7 ± 0.2 nm and 391 ± 74 nm also observed on the surface, which may suggest that these smaller units assembled to yield the larger network-like structure. At 21 days of aging, we observed a further change: the network and small flakes had evolved into much larger flakes with an average height and diameter of 2.2 ± 0.3 nm and 2.4 ± 0.3 μm.

The self-assembly of ILVAGK was altered by the presence and increasing concentration of DNA–peptide conjugates (Fig. 2b, S9–S12†). With 0.1 equivalents of A′–ILVAGK the appearance of network-like structures was now not observed until 21 days of aging. The structures prior to that point also differ from those seen in the pure ILVAGK sample, in that they appeared to be less ordered, and the network-like structure with a height between 1 and 2 nm, (21 days aging) in the 0.1 equiv. sample was more densely interlinked in comparison to the pure ILVAGK sample (14 days). Self-assembly differed again with equimolar A′–ILVAGK and ILVAGK. At 0 and 14 days of aging fibre-like structures were produced with heights between 0.5–3 nm (0 days) and 0.5–2 nm (14 days) respectively (Fig. 2c). These fibre-like structures led to the formation of a very large structure at 21 days of aging which appeared to be built up of striated sheets with a height ranging between 1 and 6 nm across the whole structure. After a further week, these structures transformed into a web-like network distinct from all other structures observed. One possibility is that the addition of DNA–peptide conjugates into the peptide system slows the overall self-assembly of ILVAGK, possibly because the DNA partially inhibits the peptide monomers from coming together because of their size, preventing formation of kinetically trapped disordered structures and instead promote more ordered, equilibrium structures. Alternatively, it may be that different kinetically trapped structures are obtained. Since the hydrophobic character of ILVAGK promotes formation of disordered gelating aggregates when heated, thermal annealing was not used to examine this possibility. Further evidence of the effect of conjugated DNA-strands was seen in the distinct fibrillation evident in the pure A′–ILVAGK sample (Fig. 2d, S8†), with a progression from elongated structures 2.8 ± 0.2 nm high extending from angular seeds, to formation of sheet-like aggregates which appear to derive from collection of the fibres (21 days) to a continuous film (28 days). This growth in consistent with the DLS data.

Study 1 therefore established that peptide driven self-assembly is altered with the addition of DNA–peptide conjugates, changing both the type of structures and the timescales on which they appear. To further explore the role of DNA within this system DNA hybridisation was introduced as another level of control which could indirectly affect the peptide-driven self-assembly.

Study 2 explored the effect of adding DNA hybridisation to the peptide-driven self-assembly. To achieve this, A–ILVAGK was mixed with DNA strands which were 1, 2, and 3 complements long (A′, 2A′ and 3A′ respectively, see ESI† for sequences). With a single complement (*i.e.* using A′), it was anticipated that the self-assembly would differ from that observed in Study 1 due to the rigidity of dsDNA relative to ssDNA, as well as by the increased density of negative charge. With multimeric-complement strands, it was expected that the DNA would either crosslink or deform the peptide assemblies, since we would have 2A′ and 3A′ each fully hybridised with two or three instances of A–ILVAGK, resulting in a DNA double helix decorated with 2 or 3 peptides, which would tend to their own arrangement. By interfacing the DNA and peptide assembly indirectly, we enabled hierarchical and emergent supramolecular behaviour. This is of interest as systems with hierarchy show unusual properties when compared with their component parts. For example, the Stupp lab have used DNA–peptide conjugates to produce structures similar to those expressed in the extracellular matrix of cells.^32^

**Fig. 3:**
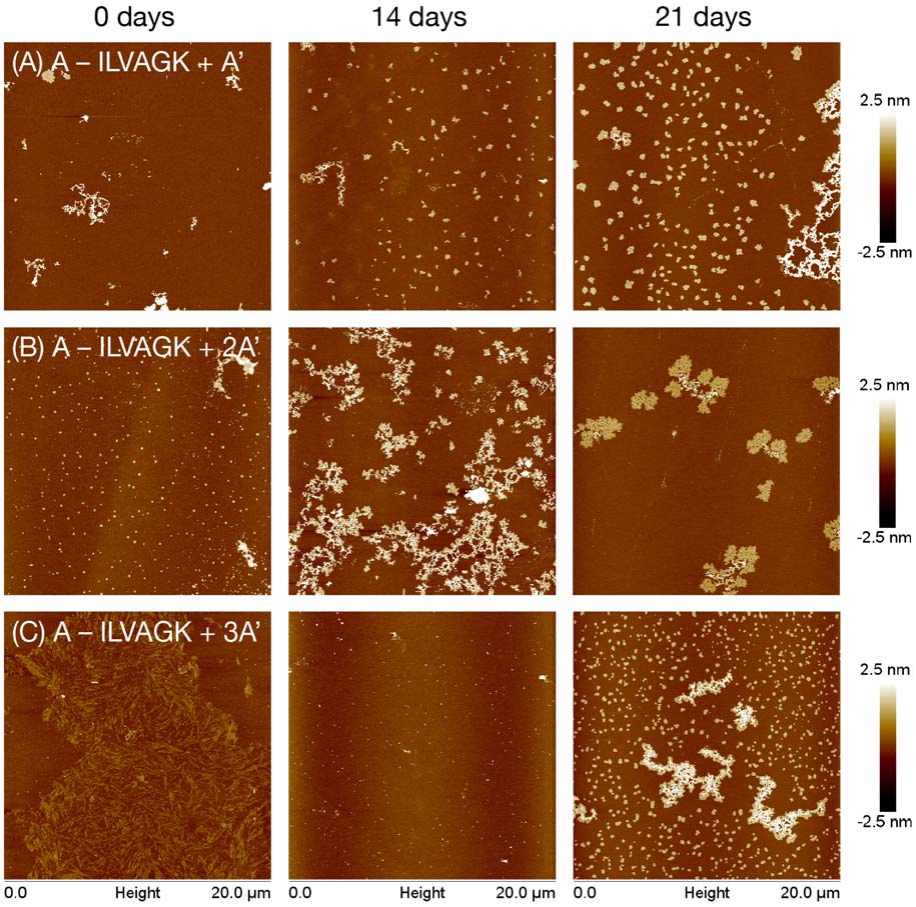
AFM images showing change in self-assembly of hybridised A–ILVAGK DNA–peptide conjugate and extended complements A′ (a), 2A′ (b) and 3A′ (c), during 28 days of aging in TAMg buffer (see also Fig. S14–S16†). Time points depicted as 0, 14 and 21 days of aging. All AFM was carried out at a concentration of 10 μM.

A–ILVAGK + A′ (1 : 1 equiv.), A–ILVAGK + 2A′ (2 : 1 equiv.) and A–ILVAGK + 3A′ (3 : 1 equiv.) were heat-cooled from 55 °C to 4 °C over 30 minutes in TAMg buffer to ensure correct hybridisation, and then aged and analysed as in Study 1. DLS measured a general increase in apparent diameter over the time period, confirming evolution progressively larger supramolecular aggregates (Fig. S14†). CD spectroscopy measured strong features for DNA and a weak peak at ∼203 nm for the peptide, and as a whole the spectra remained steady over the period (Fig. S15†), indicating good stability of the double helix and not showing distinctive changes in peptide β-turn conformation in the interior of the growing nanostructures. AFM (Fig. 3, S14–S16†) revealed that the superstructures were not so static, however. A–ILVAGK + A′ at 0 days of aging (Fig. 3a, S16†) produced a few fractal-like structure with a height of 2.5–5 nm; after 14 days of aging there were fewer of these fractal-like structures and more flakes, with a height and diameter of 2.9 ± 5.6 nm and 306 ± 75 nm respectively. At 21 days of aging, we observed a large fractal-like network (H: 1.5–3 nm) with more flakes (H: 1.9 ± 0.4 nm D: 379.3 ± 113.4 nm) visible on the surface. The appearance of flakes after 14 days of aging, may suggest the presence of a DNA double helix has improved the ability of the peptide to self-assemble, compared to the 1:1 doping of ILVAGK with A′–ILVAGK for the same time point (Fig. 2c), *i.e.* the double stranded DNA made self-assembly more closely resemble that of the un-annealed pure peptide (Fig. 2a), but hindering the tight fibrils of pure A′–ILVAGK (Fig. 2d). This may be because DNA interference with ILVAGK self-assembly was reduced by making the DNA component rigid in its double helical form. However, by hybridising two conjugates to 2A′ we then increased the complexity of the system. This was evidenced by the changes in self-assembly over the aging timeframe for A–ILVAGK + 2A′ and 3A′. Doubling the length of the DNA helix (2A′), introduced a second instance of peptide from which crosslinking or assembly could occur. At 0 days of aging for A–ILVAGK + 2A′ we observe a surface covered in small flakes (H: 4.3 ± 4.7 nm and D: 226.9 ± 4.8 nm). These structures grew into network-like self-assemblies (H: 1.5–3 nm) at 14 days of aging (Fig. 3b, S17†). At 21 days of aging these network-like structures appeared to break down into smaller fractal-like self-assemblies, with a height between 0.5–1.5 nm at the edges and 2.5–4 nm at the centre. In contrast, the self-assembly observed for A–ILVAGK + 3A′ (Fig. 3c, S18†) was distinct again. At 0 days of aging, we observed a densely covered surface with fibre-like structures, with a height between 0.5 and 1 nm. In contrast to the previous two samples, there was a breakdown into small dot-like structures at 14 days of aging, with a height between 3 and 4 nm, with growth into small flakes (H: 1.7 ± 0.4 nm and D: 284.5 ± 52.0 nm) and fractal-like self-assemblies with a height between 1 and 2.5 nm, at 21 days of aging. There are similarities between the structures at the 21 day mark for the A′ and 3A′ complexes – this in itself is not surprising since the only chemical difference between the samples is an additional two phosphodiester linkages, and nanostructures which can accommodate these changes might not require a huge rearrangement. However, the routes taken to achieve these structures are quite distinct, illustrating the importance of dynamism in DNA–peptide assembly. Furthermore, the structures at the 28 day mark (Fig. S16–S18, ESI†) show that the samples are on different trajectories. Importantly, none of the samples tended towards the same fibrillar structures observed with pure A′–ILVAGK in Study 1, showing that hybridisation was altering the range of possible nanostructural outcomes. To validate these findings, control experiments were conducted using a 1 : 1 mixture of unconjugated DNA A and ILVAGK. The AFM (Fig. S19†) showed nanostructures which tended over the course of the experiment towards long fibres which are characteristic of the peptide on its own as previously reported when annealed^26^ (and not dissimilar to those in some of the doping studies). There were also other, independent somewhat flake-like structures which are similar to controls with pure DNA (Fig. S29†), although also show some similarity to the pure peptide in TAMg (Fig. S13†).

Study 2 has shown that the addition of a rigid DNA double helix introduces a second level of self-assembly system which enhanced and changed the structures seen, previously driven only by peptidic self-association. The study highlights that seemingly well-defined structures as seen initially with 3A′ can be transient over longer timescales.

**Fig. 4:**
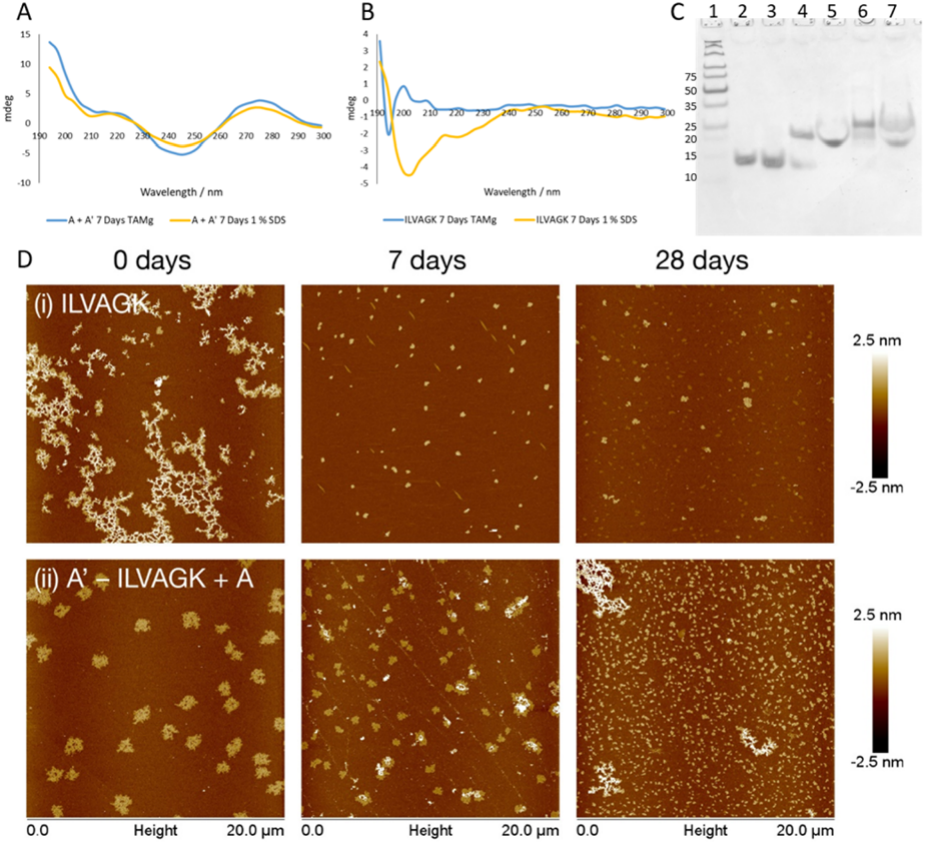
(A) CD spectra showing that DNA hybridisation of A, and A′ is maintained in both TAMg and SDS buffer conditions. (B) CD spectra showing ILVAGK conformation in TAMg and 1% SDS conditions. (C) 12% non-denaturing PAGE (1) ladder, (2) A. (3) A′, (4) A + A′ hybridised in TAMg, (5) A + A′ hybridised in 1% SDS, (6) A′–ILVAGK + A hybridised in TAMg, (7) A′–ILVAGK + A hybridised in 1% SDS. (D) AFM images showing change in self-assembly of ILVAGK (i) and hybridised A′–ILVAGK DNA–peptide conjugate (ii) in 1% SDS, over 28 days of aging. Time points depicted as 0, 7 and 28 days of aging. All AFM, CD and PAGE were carried out at a peptide/DNA concentration of 10 μM.

Study 3 was designed to clarify orthogonality present in our DNA–peptide conjugates. Orthogonality of self-assembly within synthetic systems is currently of interest since it allows more sophisticated mimicry of nature.^33^ If our system exhibits orthogonal behaviour, this opens up opportunities for nanoscale controllability and complexity previously seen only in biology.^2^ Study 2 illustrated that we can control DNA-directed assembly by adding (or not adding) the appropriate complementary strands. By arresting the peptide self-assembly pathway, while retaining the DNA hybridisation, we should be able to establish whether we have achieved orthogonality. A buffer system which contained enough metal ions to support the formation of a DNA double helix but did not promote the self-assembly of ILVAGK was needed, and a 1% sodium dodecyl sulphate (SDS) aqueous solution was identified as a suitable medium.^34^ DLS of ILVAGK and A′–ILVAGK in 1% SDS (Fig. S20†) showed that self-assembly was minimal and static over 28 days. Fig. 4a displays the CD spectra of the A/A′ double helix in both TAMg and 1% SDS buffer systems; it can be seen from the close similarity of these spectra that the double helix is intact in both buffer systems. In contrast, Fig. 4b has the CD spectra of ILVAGK in both buffer systems; it shows that ILVAGK adopts a β-turn formation in TAMg buffer, but is a random coil in 1% SDS buffer. These CD spectra are stable over the full 28 day aging period (Fig. S21†). Polyacrylamide gel electrophoresis (PAGE, Fig. 4c) of samples prepared in 1% SDS confirmed the presence of the DNA double helix in both the A + A′ (lane 5) and A′–ILVAGK + A (lane 7) combinations, with the same mobility as the SDS-free samples (lanes 4 and 6 respectively). The lack of higher molecular weight bands in Fig. 4c is also suggestive of the success in preventing the self-assembly of ILVAGK. AFM imaging (Fig. 4d, S22 and S23†) showed that at 0 days of aging in 1% SDS, ILVAGK (Fig. 4d(i)) gave self-assembly similar to the network-like structures (H: 1–2 nm) seen in Study 1. This may be due to some self-assembly being established before treatment with SDS. The low percentage of SDS prevents further self-assembly of peptides but is less effective at breaking down already assembled structures. Over the 28 days aging period we saw reduced nanostructural evolution: there were small flake structures with an average height and diameter of 3.1 ± 7.2 nm and 296.9 ± 54.9 nm at 7 days of aging, with some further growth in diameter at 28 days of aging to an average height and diameter of 1.8 ± 0.6 nm and 363.9 ± 100.8 nm, respectively.

This would suggest a breakdown in the established self-assemblies by 7 days of aging and a stable self-assembly reached after this point. In contrast, A′–ILVAGK + A (Fig. 4d(ii)) at 0 days of aging we observed large flakes (H: 1.5 ± 0.1 nm and D: 1.0 ± 0.1 μm), which at 7 days of aging shrank in height to an average of 1 nm for the flatter flakes with an average diameter of 505.2 ± 234.3 nm and an average height of 18.1 ± 12.4 nm for the layered flakes. These structures further devolved to small flakes at 28 days of aging, with an average height and diameter of 1.8 ± 1.8 nm and 282.5 ± 120.6 nm, respectively (Fig. 4d(ii)). Although the size of these structures did change their morphology is alike. We believe that this lack of diverse self-assembly when compared to that observed in Study 2 (A–ILVAGK + A′, Fig. 3a), shows that we can prevent or allow each self-assembly mechanism to progress independently, giving orthogonality. Since the DNA- and peptide-driven associations do not directly interact, the new structures arising from Study 2 are likely to arise from hierarchical or emergent assembly. Study 3 has shown that we are able to halt or promote peptide self-assembly using SDS, as well as introduce DNA self-assembly by the presence or absence of DNA complements. This control has also allowed us to confirm the presence of hierarchical self-assembly within our system.

## Conclusion

By combining a self-assembling peptide with an oligonucleotide capable of hybridising with its complementary strand, we have created a doubly functional biomacromolecular hybrid. We have shown that the supramolecular chemistry of peptide and oligonucleotide segments can be operated independently, and thus the system displays orthogonality. This is not to say that the two regimes do not influence each other – presence of the oligonucleotide segment changes the rate of formation and eventual outcome of the resultant nanostructures. Moreover, when both peptide and oligonucleotide assembly are operational the kinetics and thermodynamics of the system are again changed as these two systems interact indirectly (*i.e.*, not through direct molecular contact of peptide and DNA fragments). Such indirect effects can be called ‘emergent’ in the sense that they operate only at the level of the conjugates, rather than at the level of the individual DNA and peptide components. Emergent assembly like this is widespread in nature, and this system provides an opportunity to further explore the possibilities of more nature-like self-assembly of functional nanostructures. The dynamic component of this work is also important: peptide and DNA nanostructures can be dynamic, even after annealing, and it is important that future development of these technologies take this aspect into account.

## Data availability

The ESI† contains experimental details, NMR and MS data, additional AFM images, DLS, and CD spectra. Unprocessed spectroscopic and AFM data are available from the authors upon request.

## Author contributions

Emerald R. Taylor conducted the bulk of the experimental work, analysed the data, and drafted the manuscript. Akiko Sato and Isobel Jones contributed to the synthesis and characterisation of the conjugates. Prashant G. Gudeangadi and David M. Beal contributed to conjugation strategies. James A. Hopper helped analyse the CD spectra. Wei-Feng Xue collected a portion of the AFM data and contributed to overall analysis and presentation of AFM. Michael R. Reithofer and Christopher J. Serpell conceived and supervised the project. All authors have edited and approved the manuscript.

## Conflicts of interest

None to declare.

## Supplementary Material

SC-014-D2SC02482A-s001
