# Supplementary material for: Tuning dynamic DNA- and peptide-driven self-assembly in DNA–peptide conjugates
Source: Chem Sci. 2022 Nov 30;14(1):196–202. doi: 10.1039/d2sc02482a (PMC9769108; doi:10.1039/d2sc02482a)
Supplement: SC-014-D2SC02482A-s001 [file SC-014-D2SC02482A-s001.pdf]

Electronic Supplementary Material (ESI) for Chemical Science.
This journal is © The Royal Society of Chemistry 2022

Tuning Dynamic DNA and peptide driven self-assembly in DNA-peptide conjugates.

Emerald R. Taylor,[a] Akiko Sato,[a] Isobel Jones,[a] Prashant G. Gudeangadi,[a] David M. Beal,[b] James A. Hopper,[a] Wei-Feng Xue,[b] Michael R. Reithofer*[c] and Christopher J. Serpell*[a]

[a] School of Chemistry and Forensic Science, Ingram Building, University of Kent, Canterbury, Kent, CT2 7NH, UK
[b] School of Biosciences, Stacey Building, University of Kent, Canterbury, Kent, CT2 7NJ, UK
[c] Dept. of Inorganic Chemistry, University of Vienna, Währinger Straße. 42, 1090 Vienna, Austria

Supplementary Information

Contents

Materials. Instrumentation and general considerations 2

Synthesis and characterisation of DNA-peptide conjugates 6

Study 1: DLS, CD and AFM 8

Study 2: DLS, CD and AFM 16

Study 3: DLS, CD and AFM 22

Control samples 25

Materials, instrumentation, and general considerations

Dry acetonitrile, urea, EDTA, stains all gel stain, formamide and 6-maleimidohexanoic acid were purchased from Acros. Acrylamide/bisacrylamide 37.5:1 solution; dichloromethane, potassium carbonate anhydrous, hydrochloric acid, sodium chloride, sodium dodecyl sulfate, tris base, magnesium acetate tetrahydrate, glacial acetic acid, boric acid and *N*-hydroxy succinimde were purchased from Fisher Scientific. Ammonium phosphate, *N*, *N, N'*, *N'*-tetramethylethylenediamine (TEMED) and (1R,8S,9s)-Bicyclo[6.1.0]non-4-yn-9-ylmethyl *N*-succinimidyl were purchased from Sigma Aldrich. *N*-(3-Dimethylaminopropyl)-*N'*-ethylcarbodiimide hydrochloride (EDC.HCl) and *N*, *N*-diisopropylethylamine were purchased from Fluorochem. Zetadex-25 medium was purchased from emp Biotech. Mica discs, 10 mm were purchased from agar scientific. Q Sepharose fast flow, 25 mL was purchased from GE healthcare. All materials were used as provided, without further purification unless otherwise stated.

DNA strands were purchased from Integrated DNA Technologies. The strand sequences were as follows:

Table S1. Table showing DNA sequences and properties used within this work.

Name	Sequence	Length	Tm (˚C)
A	CTG TAT GGT CAA CTG	15*mer*	42
A-T5	TT TTT CTG TAT GGT CAA CTG	20*mer*	42
A-NH_2	NH_2 - TT TTT CTG TAT GGT CAA CTG	20*mer* + C_6 - NH_2	42
A'	CAG TTG ACC ATA CAG	15*mer*	42
A'-T5	TT TTT CAG TTG ACC ATA CAG	20*mer*	42
A'-NH_2	NH_2 - TT TTT CAG TTG ACC ATA CAG	20*mer* + C_6 - NH_2	42
2A'	CAG TTG ACC ATA CAG CAG TTG ACC ATA CAG	30*mer*	42
3A'	CAG TTG ACC ATA CAG CAG TTG ACC ATA CAG CAG TTG ACC ATA CAG	45*mer*	42

TAMg buffer is composed of 45 mM Tris and 12.5 mM $Mg(OAc)_2 \cdot 6H_2O$ with pH adjusted to 8.0 using glacial acetic acid. TBE buffer is 90mM Tris, 90mM boric acid and 1.1mM EDTA with a pH of 8.0. Denaturing PAGE is conducted in the presence of 2.4 M urea.

Dynamic light scattering (DLS): Samples were prepared in TAMg or 1 % SDS buffer (1 mL, 10 µM). A 200 µL aliquot was extracted and measured at 0, 7, 14, 21 and 28 days of aging for studies 1 and 2. For study 3 an aliquot was measured at 7 and 28 days of aging. The missing data points were due to Covid-19 related isolation and instrument malfunction (0 days aging). Analysis was carried out on a Malvern Zeitasizer Nano ZS. The sample was equilibrated to 25 °C before 3 runs of 11 consecutive scans of the sample were taken.

Atomic Force Microscopy (AFM): Samples were prepared in TAMg and 1 % SDS buffer (1 mL, 10 µM). Mica was cleaved using PVC tape until a smooth surface was achieved immediately prior to use. Sample was deposited onto the freshly cleaved mica (20 µL of 10 µM). The samples were incubated for 30 minutes at room temperature, then carefully dried off using filter paper. The surfaces were then carefully washed with 750 µL filtered autoclaved H_2O at an angle, excess water was removed using filter paper and samples were subsequently dried under gentle stream of nitrogen for 2 minutes. Samples were stored in a petri dish with lid at room temperature. Images were acquired on a Bruker Multi-Mode microscope with a Nanoscope 8 controller operating under ScanAsyst mode in air. Bruker ScanAsyst-Air cantilever tips with nominal tip radius of 2 nm and spring constant of 0.4 N/m were used. Images were flattened and scan tilt and bow were removed using Nanoscope Analysis 1.5 software (Bruker, CA, US).

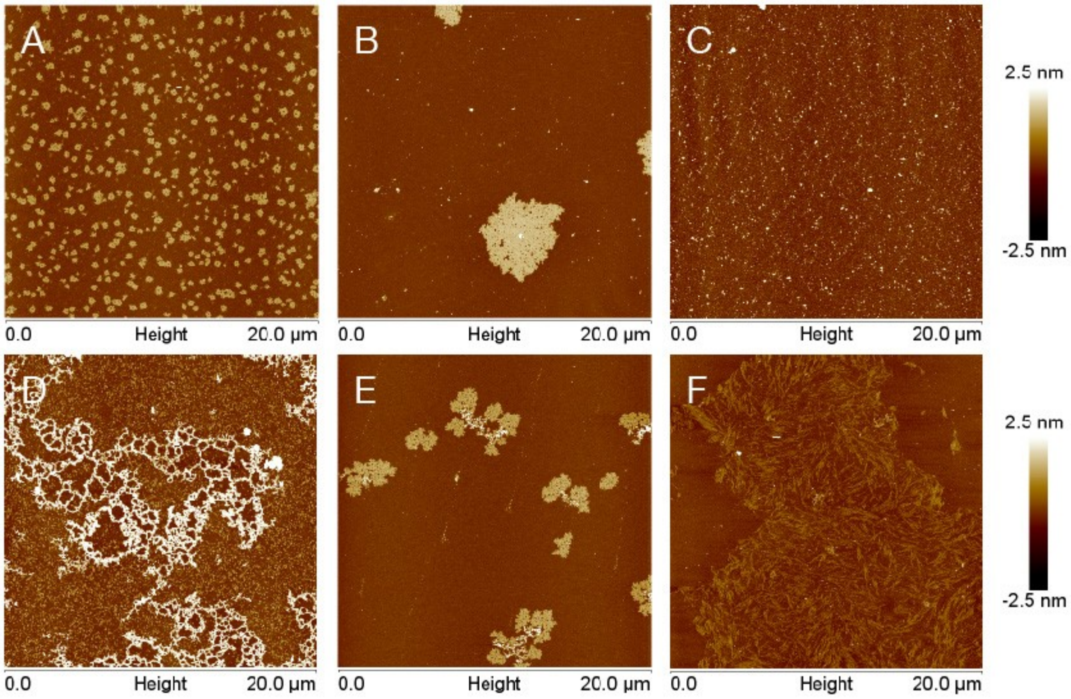

Figure S1. Morphologies within this paper. A: Small flakes, B: large flakes, C: dot-like, D: network-like, E: fractal-like and F: fibre-like.

Circular Dichroism (CD): Samples were prepared in TAMg or 1 % SDS buffer (1 mL, 10 µM). A 200 µL aliquot was extracted and measured at 0, 7, 14 and 35 days of aging for studies 1 and 2. For study 3 an aliquot was measured at 0, 7 and 28 days of aging. The missing data points were due to Covid-19 related isolation. Circular dichroism measurements were recorded using a Jasco J-715 spectropolarimeter, with temperature control provided by a NesLab RTE-111 circulating chiller operating at 20 ± 0.05 °C. Spectra were recorded in a 0.2 cm pathlength quartz cell, maintaining consistent cell orientation. Spectra were averaged over 20 readings, and subsequently smoothed using 3-point median smoothing.

Mass Spectrometry: Electrospray LC-MS of Oligonucleotides. Electrospray mass spectra were recorded on a Bruker micrOTOF-Q II mass spectrometer. Samples were introduced into the mass spectrometer by on-line reverse-phase HPLC on a Phenomenex Nucleosil C18 column (3 µm, 120Å, 2.0 mm x 150 mm) running on an Agilent 1100 HPLC system at a flow rate of 0.2 ml/min using a short gradient from 10% B to 100% B (A: 15 mM TEA, 400 mM HFIP in water; B: 15 mM TEA, 400 mM HFIP in methanol). The eluent was monitored at 200-800 nm and then directed into the electrospray source, operating in negative ion mode, at 3.5 kV and mass spectra recorded from 400-3000 m/z. Data was analysed and deconvoluted to give uncharged oligonucleotide masses with Bruker's Compass Data Analysis software.

Denaturing Polyacrylamide Gel Electrophoresis (PAGE): A 5 mL 20 % denaturing polyacrylamide gel was prepared from a 20 % acrylamide / Bis-acrylamide denaturing gel stock solution. To this 5 µL of TEMED and 10 µL of 40 % APS stock solution was added and mixed well. The gel was added to the cassette and a 10 well comb inserted, the gel was left to polymerise. Samples were prepared from DNA-peptide conjugates in water (10 µL, 20 µM) and 8 M aqueous urea (10 µL). Samples were mixed well and centrifuged to ensure the sample was in the bottom of the tube. Once polymerised the well comb was removed from the cassette and the samples loaded (10 µL per well). The gel was placed into the inner chamber and placed within the gel tank. 1 x TBE buffer was added to the inner and outer chamber. The gel ran for 60 minutes at maximum volts and 15 mAmps. The gel was removed from the cassette and stained in Stains All gel stain (0.05 mg / mL in 50 : 50, formamide : 1 x TBE buffer) for a minimum of 1 hour. The gel was imaged on a document scanner. Images were enhanced using GIMP 2.10.8 imaging software, only to enhance contrast and colour.

Non-denaturing Native Tris Magnesium Acetate Polyacrylamide Gel Electrophoresis (TAMg PAGE): A 5 mL 12 % non-denaturing polyacrylamide gel was prepared from a 12 % acrylamide / Bis-acrylamide gel stock solution

(5 mL). Different concentrations can be prepared by diluting the stock with the relevant amount of buffer. To this 5 µL of TEMED and 10 µL of 40 % APS stock solution was added and mixed well. The gel was added to the cassette and a 10 well comb inserted, the gel was left to polymerise. 10 µL of sample (20 µM) was mixed with its complement (20 µM) and 5 µL of glycerol. This was repeated for each sample. Controls were prepared from stocks of the individual oligomers (10 µL, 20 µM) without their complements and glycerol (10 µL). All samples were heat cooled using a thermocycler using the following programme: Heated to 55 °C for 5 minutes then cooled to 35 °C for 30 minutes, and finally cooled to 4 °C and held there for 1 hour. 10 µL of each sample was loaded onto the gel. The gel ran at maximum volts (300 V), constant mAmps (15 mAmps per gel) for 90 minutes in 1 x TAMg buffer. The gel was removed from the cassette and stained in Stains All gel stain (0.05 mg / mL in 50 : 50, formamide : 1 x TAMg buffer) for a minimum of 1 hour. The gel was imaged on a document scanner. Images were enhanced using GIMP 2.10.8 imaging software, only to enhance contrast and colour.

Synthesis of ILVAGK Azido Peptide:

Table S2. Table showing Amino acid sequence and abbreviations.

Amino Acid	3-letter Abbreviation	Single letter Abbreviation
Isoleucine	Ile	I
Leucine	Leu	L
Valine	Val	V
Alanine	Ala	A
Glycine	Gly	G
Lysine	Lys	K

N_3-ILVAGK-NH_2 was synthesized following standard solid-phase peptide synthesis protocols. In short, Fmoc-Lys(Boc)-OH (2. equiv.) was immobilized on Rink amide resin using TBTU, HOBt, and DIPEA (2. equivalent each) in DMF. The resulting reaction was allowed agitated for 2 h after which any free amino group was blocked using acetic anhydride and pyridine (1 mL/ 1g resin). Following washing with 5 x DMF, the Fmoc group was removed using a solution of 20% piperidine in DMF. Subsequently, the peptide was assembled using appropriate Fmoc-protected amino acid following standard coupling procedures. After the full peptide has been assembled, azidopropinoic acid was coupled using TBTU, HOBt, and DIPEA. The completeness of the coupling was tested using a Kaiser test, and if needed the azidopropinoic acid was recoupled until a Kaiser test was negative. The fully assembled peptide on the resin was washed with DMF and DCM and dried in vacuum. Subsequently, the peptide was cleaved using 95% TFA, 2.5% TIS, and 2.5% H_2O as cleaving cocktail. The final peptide was obtained after precipitation with cold diethyl ether. The peptide was analysed by ESI mass spectrometry in positive mode, with a mass of 696.443 m/z $[M + H^+]^+$. The ^1H NMR showed all the protons peaks of N_3-ILVAGK-NH_2, with proton integration similar to theoretical values (Fig. S2).

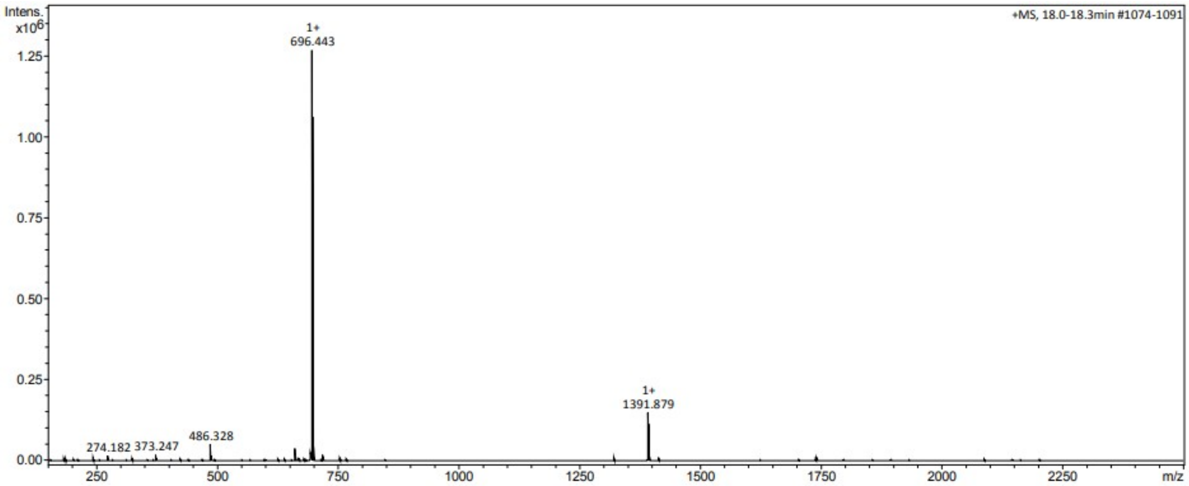

Figure S2. Deconvoluted mass spectrum of N_3-ILVAGK-NH_2. Calculated for $C_{31}H_{57}N_{11}O_7$ 695.452, found 696.443 $[M + H^+]^+$.

^{1}H NMR (400 MHz, DMSO-d_6), δ Shift ppm 0.70-0.82 (m, CH_3, [Ile, Leu, Val], 18H), Shift ppm 0.96-1.06 (m, CH, [Leu], 1H), Shift ppm 1.15 (d, CH_3, [Ala], 3H), Shift ppm 1.18-1.28 (m, CH_2, [Ile], 2H), Shift ppm 1.30-1.56 (m, CH_2, [Lys], 6H), Shift ppm 1.56-1.70 (m, CH_2, [Leu], 2H), Shift ppm 1.83-1.93 (m, CH, [Ile], 1H), Shift ppm 2.31-2.41 (m, CH, [Val], 1H), Shift ppm 2.65 (t, J=7.55 Hz, CH_2, [Lys], 2H), Shift ppm 3.39-3.45 (m, CH_2, CH_2-N_3, 4H), Shift ppm 3.66 (d, J=6.33 Hz, CH_2, [Gly], 2H), Shift ppm 4.04-4.30 (m, αC-H, 6H), Shift ppm 6.97 (s, CONH, [C-terminus], 1H), Shift ppm 7.30 (s, CONH, [C-terminus], 1H), Shift ppm 7.57 (d, J=8.89 Hz, NH, 1H), Shift ppm 7.81 (d, J=7.91 Hz, NH, 1H), Shift ppm 8.01-8.10 (m, NH, 3H), Shift ppm 8.34 (s, NH, [Gly], 1H).

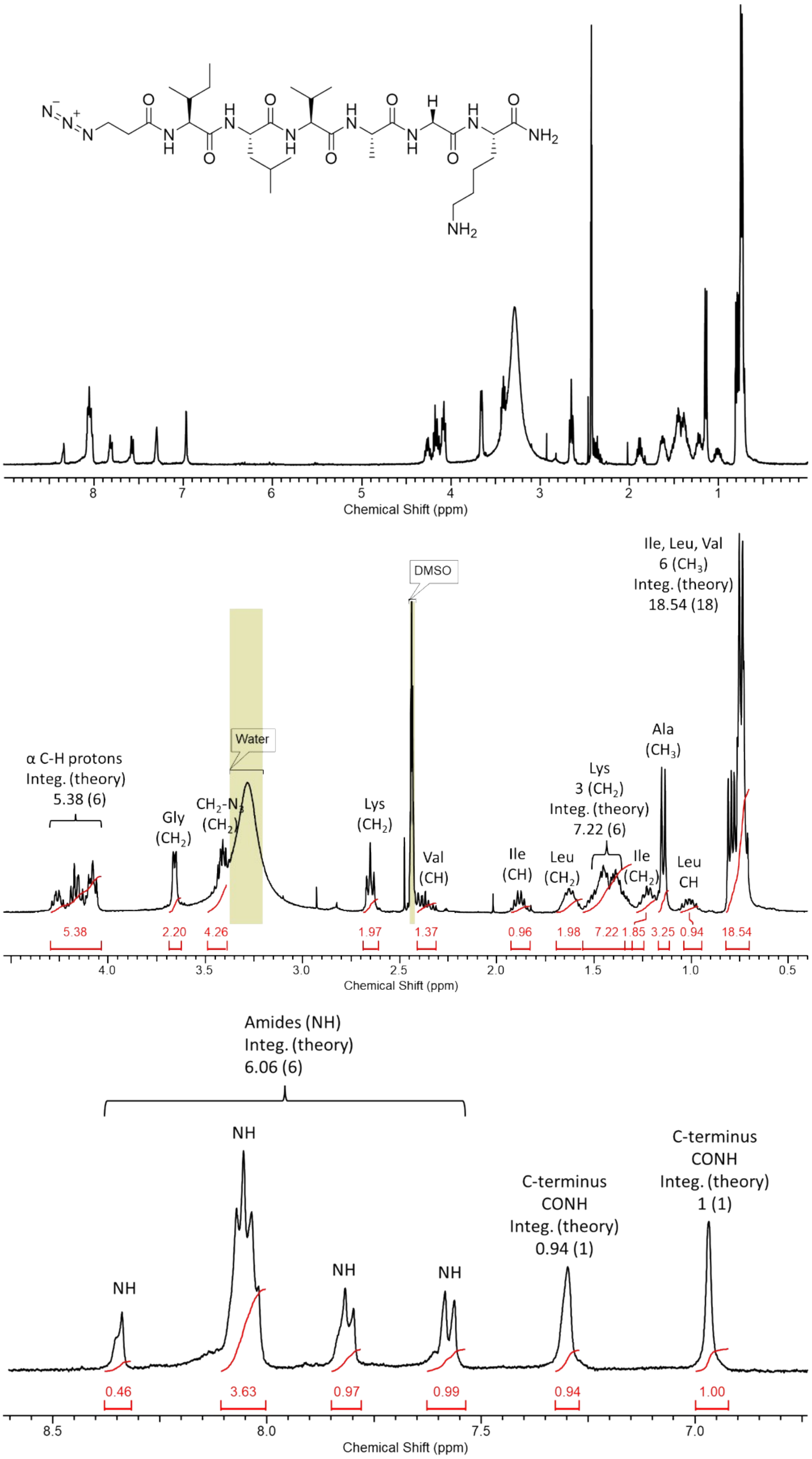

Figure S3. ^{1}H NMR of **N_3-ILVAGK-NH_2**.

Synthesis and purification of A-ILVAGK and A'-ILVAGK DNA-peptide conjugates

A 400 µL 100 µM **A-CO** solution was used from a 1 mM stock. A 200 µL 1 mM solution of azide peptide (**N_3-ILVAGK-NH_2**) in filtered autocalved water was added to **A-CO**. This was repeated for **A'-CO**. The solutions were vortexed and centrifuged in a mini centrifuge for 5 seconds, to ensure homogeneity of the solution and collect the total volume into the bottom of the tube. The oligonucleotide-peptide solutions were split into 200 µL aliquots and incubated at 37 °C for 60 minutes in a thermocycler. DNA-peptide conjugates were purified by anion exchange resin and size exclusion gel chromatography. 250 µL of anion exchange resin in 20 % EtOH was added to a centrifuge tube. The resin was centrifuged at 14800 RPM for 1 minute to collect the resin into the bottom. The supernatant was then removed and discarded. To this 1000 µL of autoclaved water was added, mixed and centrifuged at 14.8 RPM for 1 minute. The supernatant was removed and discarded, then repeated three more times. To the clean resin 800 µL of crude **A-ILVAGK** and 200 µL urea was added. The resin was incubated at RT for five minutes, then centrifuged at 14800 RPM for 1 minute and the supernatant collected into fraction 1. The resin was washed with 8M Urea and centrifuged at 14800 RPM for 1 minute. The supernatant was collected as fraction 2, the above step was repeated once more and collected as fraction 3. A millilitre of autoclaved water was added to the resin, inverted, and centrifuged at 14.8 RPM for 1 minute. The supernatant was collected and labelled as fraction 4. This was repeated once more, and these fractions analysed by UV-Vis for presence of peptide (210 nm). If peptide was still detected the above step was repeated until the reading at 210 nm was below an abs value of 0.5. 1000 µL of 0.6 M NaCl in autoclaved water was added to the resin and incubated overnight at 4°C. The resin was brought to room temperature, then centrifuged at 14800 RPM for 1 minute, the supernatant was collected and labelled fraction 6. The previous was repeated 4 times and collected as fractions 7 to 10 until DNA was no longer being eluted by UV-Vis (260 nm). This method was repeated for **A'-ILVAGK** DNA-peptide conjugates. DNA-peptide conjugates were further purified by reverse phase gel chromatography. A 20 mL syringe was prepared as a mini column by placing approximately 1 mL of cotton wool into the bottom of the syringe, the cotton wool was compacted using a spatula. Zetadex resin was mixed with deionised water to produce a slurry. The column was filled to the 20 mL mark with the zetadex slurry, and the excess water was collected into a beaker. The column was flushed with 60 mL of deionised filtered water to remove any ethanol from the column. UV-Vis quantification with Thermo Scientific™ NanoDrop™ One Spectrophotometer was taken of a control fraction to ensure a baseline reading was achieved before loading the column (no absorbance). The column was washed with 1 mL of filtered deionised water, each being collected as a new fraction. This was repeated until all DNA-peptide conjugate was eluted from the column and salt concentration monitored by UV-Vis. Any fractions containing salt were kept separately. The column was then changed, and the same process as above was repeated for the next sample. The fractions were freeze dried for 5 hours and re-suspended in a total of 1 mL filtered autoclaved water to produce a stock solution. Crude samples were analysed by electrospray ionisation in negative mode, DNA–peptide conjugates had a mass of 7148.69 $[M - H]^-$ and 7129.72 $[M-H]^-$ for **A–ILVAGK** and **A'–ILVAGK**, respectively.

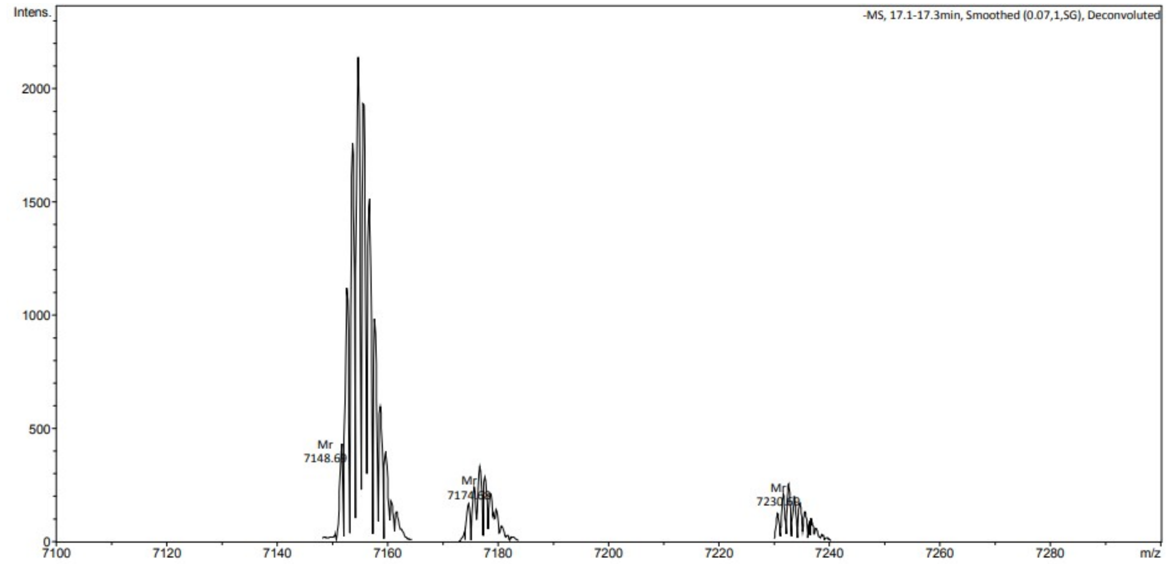

Figure S4. Deconvoluted mass spectrum of crude **A-ILVAGK**. Calculated for $[M - H]^-$ 7150.62 m/z, found 7148.69 m/z.

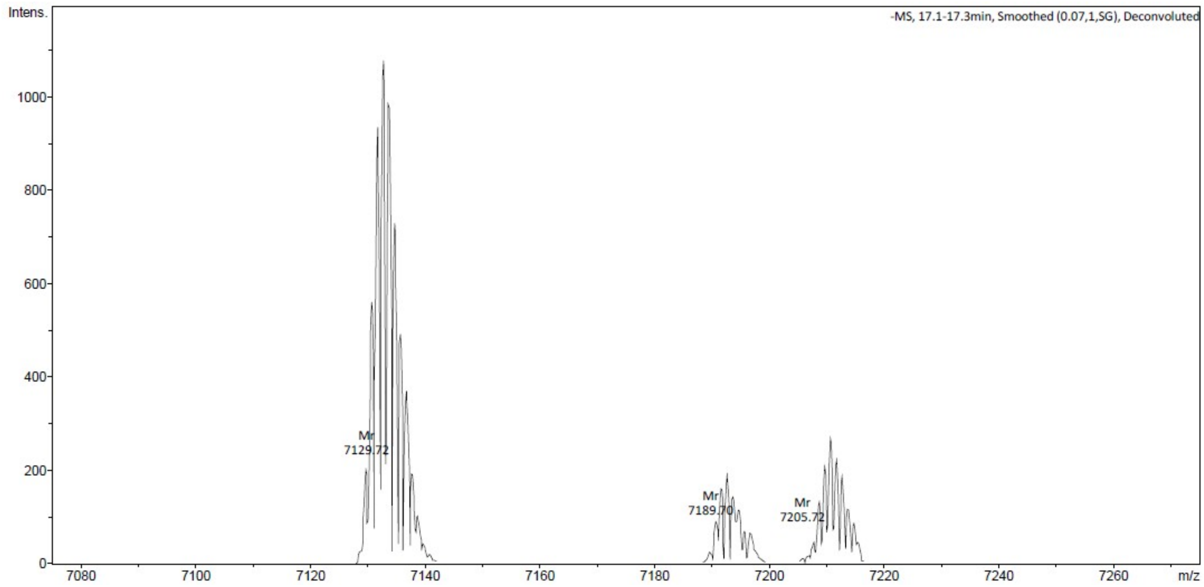

Figure S5. Deconvoluted mass spectrum of crude **A'-ILVAGK**. Calculated for $[M - H]^-$ 7128.64 m/z, found 7129.72 m/z.

Study 1

Sample preparation: A sample of 20 µM **ILVAGK** in 500 µL of filtered TAMg was prepared by diluting a 1 mM (10 µL) stock of **ILVAGK** with 490 µL of filtered TAMg buffer. A 20 µM solution of **A-ILVAGK** was prepared from a stock (42.5 µM, 235.3 µL) diluted to 500 µL with filtered TAMg buffer. The two were combined to give the 100 % (1:1) 10 µM solution. This method was repeated to yield 50 % (1:0.5, 117.7 µL **A-ILVAGK** stock), 10 % (1:0.1, 23.5 µL **A-ILVAGK** stock) and 1 % (1:0.01, 2.4 µL **A-ILVAGK** stock). Samples were stored at room temperature; in the dark and in 200 µL aliquots. Aliquots were extracted at 0, 7, 14, 21 and 28 days of aging for AFM, aliquots were further analysed by DLS and CD.

Control sample preparation: A 500 µL 20 µM solution of **A-T5** was prepared from a 100 µM stock by diluting 100 µL of **A-T5** with 400 µL of TAMg buffer. A 500 µL 20 µM solution of **A'-T5** was prepared from a 100 µM stock by diluting 100 µL of **A'-T5** with 400 µL of TAMg buffer solution. The two were combined and heat-cooled in a thermocycler by the following program: 55 °C for 10 mins, 35 °C for 20 mins, cool to 4 °C. Two 1000 µL, 10 µM samples were prepared from 100 µM stocks by diluting 50 µL with 950 µL of TAMg buffer. A 10 µM sample of **ILVAGK** was prepared from a 1 mM stock by diluting 10 µL with 990 µL of TAMg buffer. Samples were stored at room temperature; in the dark and in 200 µL aliquots. Aliquots were extracted at 0, 7, 14, 21 and 28 days of aging for AFM, aliquots were further analysed by DLS and CD.

DLS:

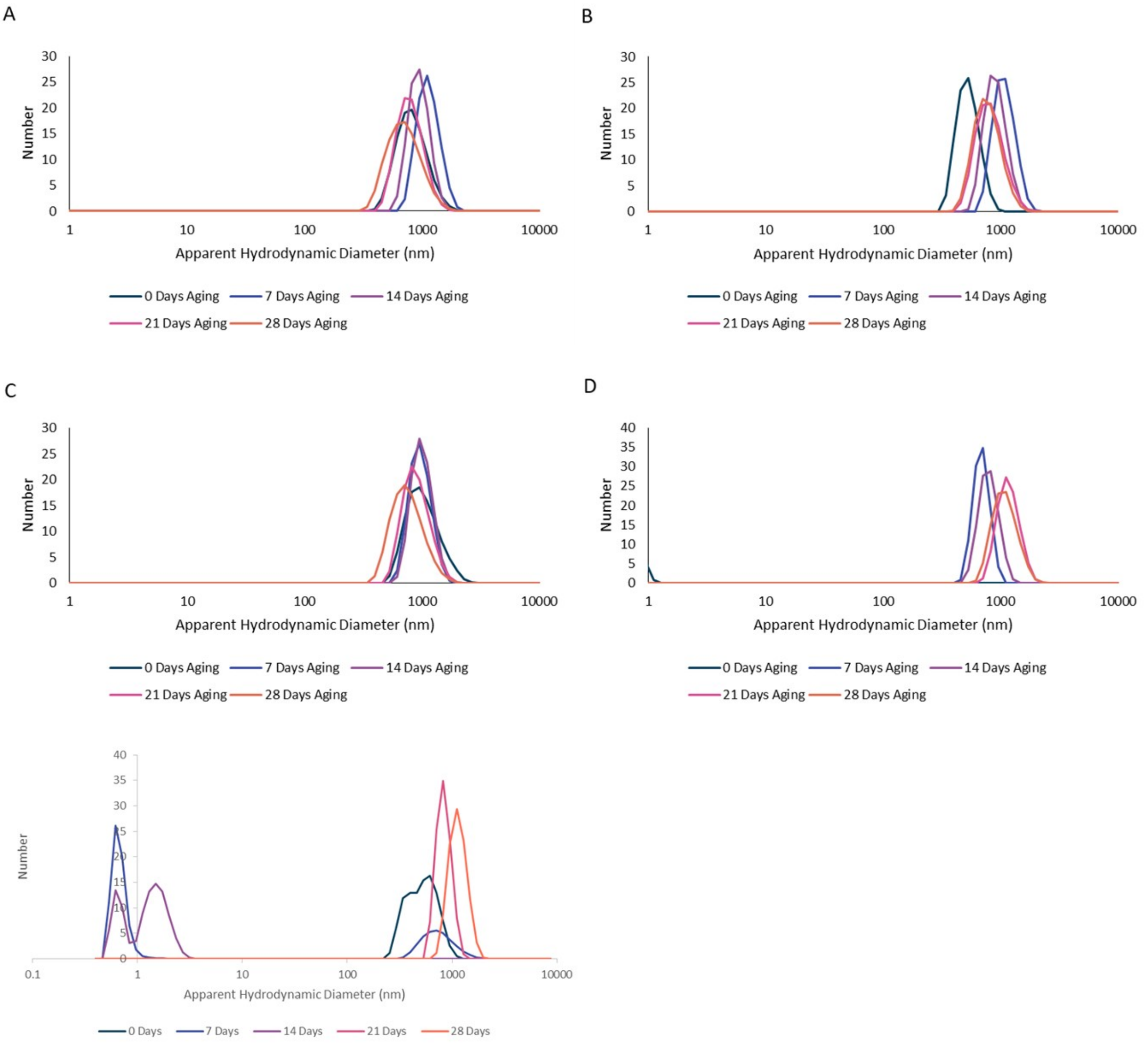

Figure S6. DLS data for Study 1. A: 1:1, B: 1:0.5, C: 1:0.1, D: 1:0.01, and E: 0:1 ratios of **ILVAGK**:**A-ILVAGK**. All measured in TAMg buffer and at a concentration of 10 μM.

CD:

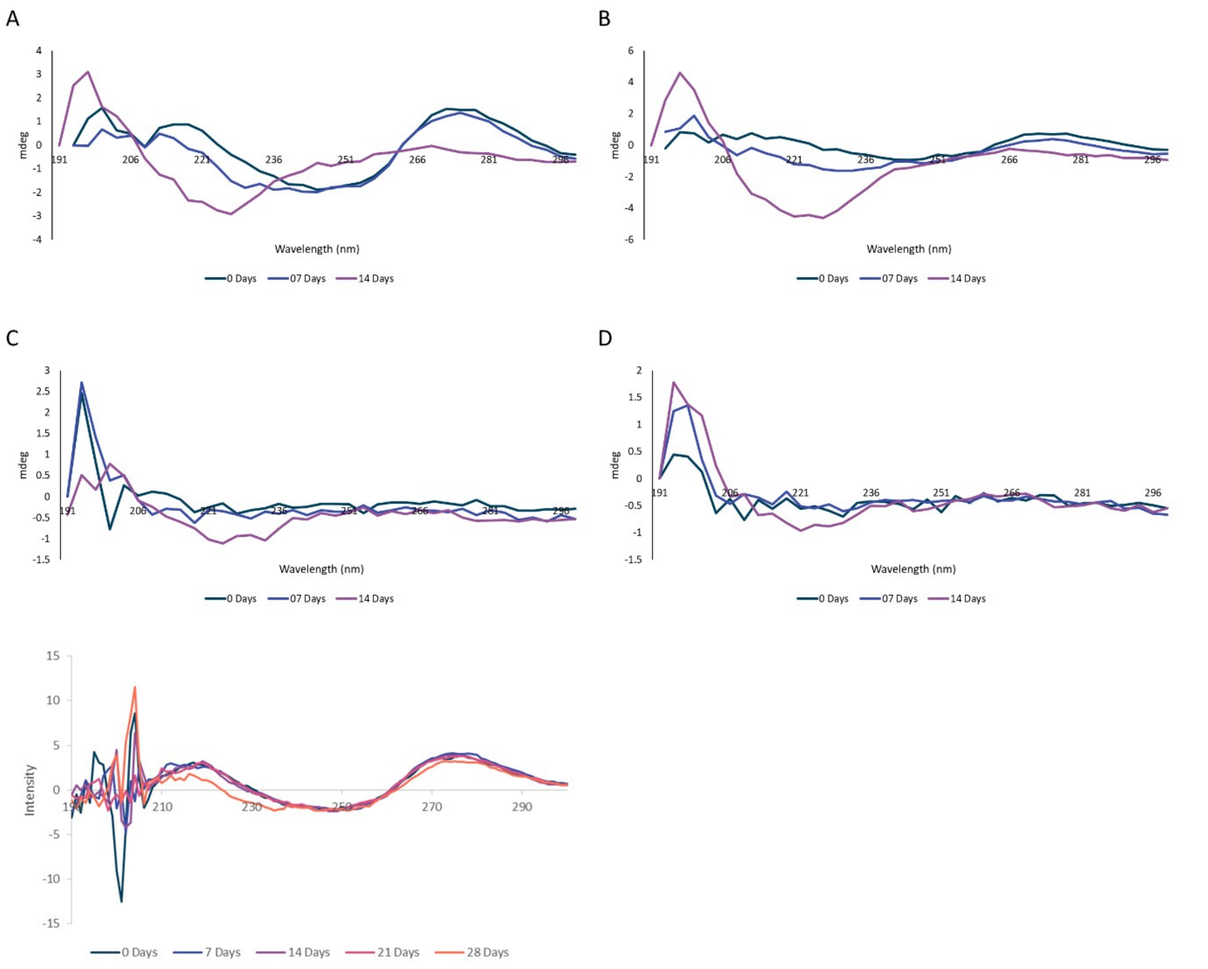

Figure S7. CD spectra for Study 1, A: 1:1, B: 1:0.5, C: 1:0.1, D: 1:0.01, E: 0:1 ratios of **ILVAGK**:**A'-ILVAGK**. All measured in TAMg buffer and at a concentration of 10 µM. Missing time points occurred due to Covid-19 related lab closures. Excessive absorption was observed with the pure **A'-ILVAGK** sample, leading to noise in the CD spectrum below 210 nm which occludes any real signal in that range.

AFM:

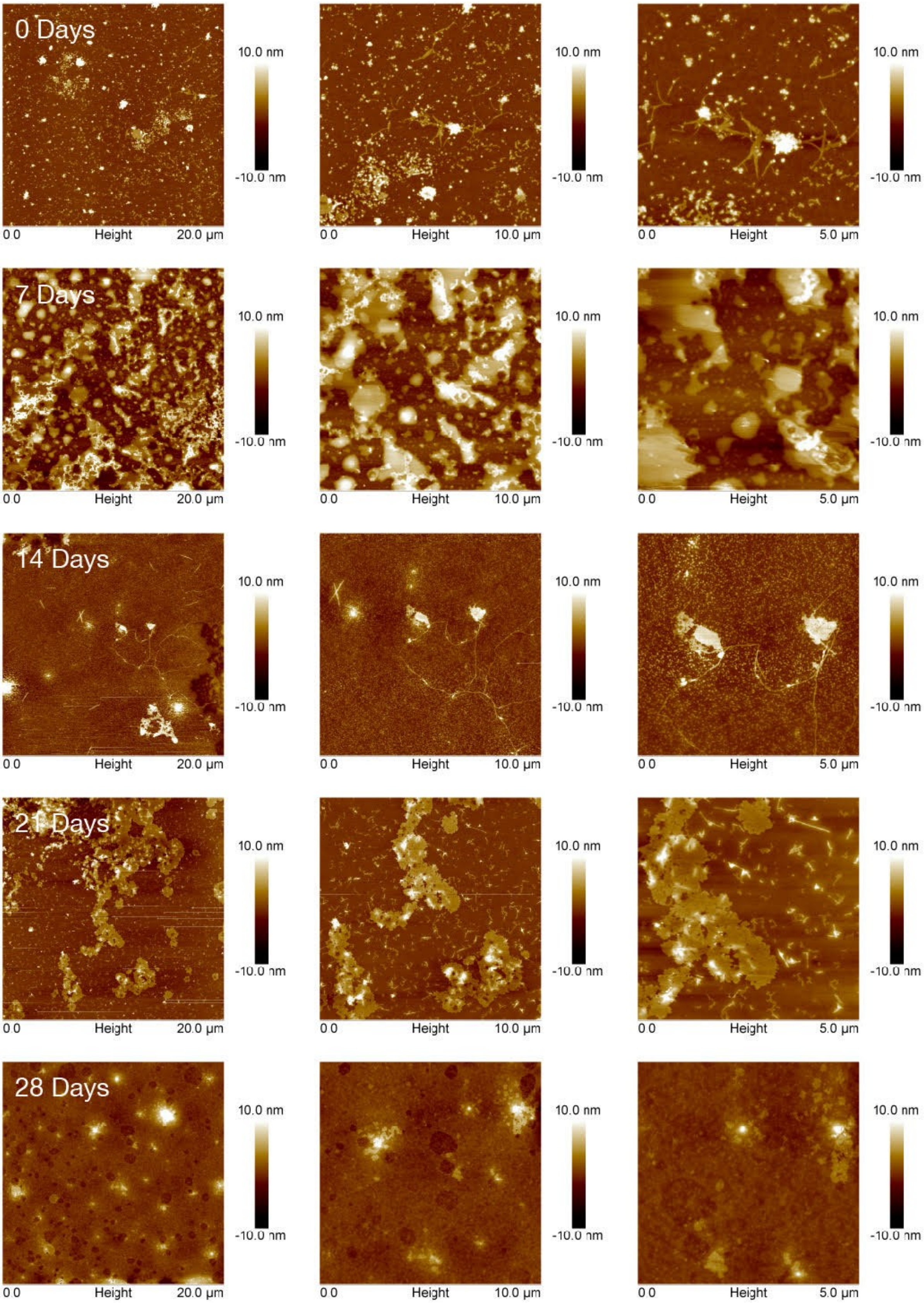

Figure S8. AFM images of pure **A'-ILVAGK** in TAMg over 28 days of aging.

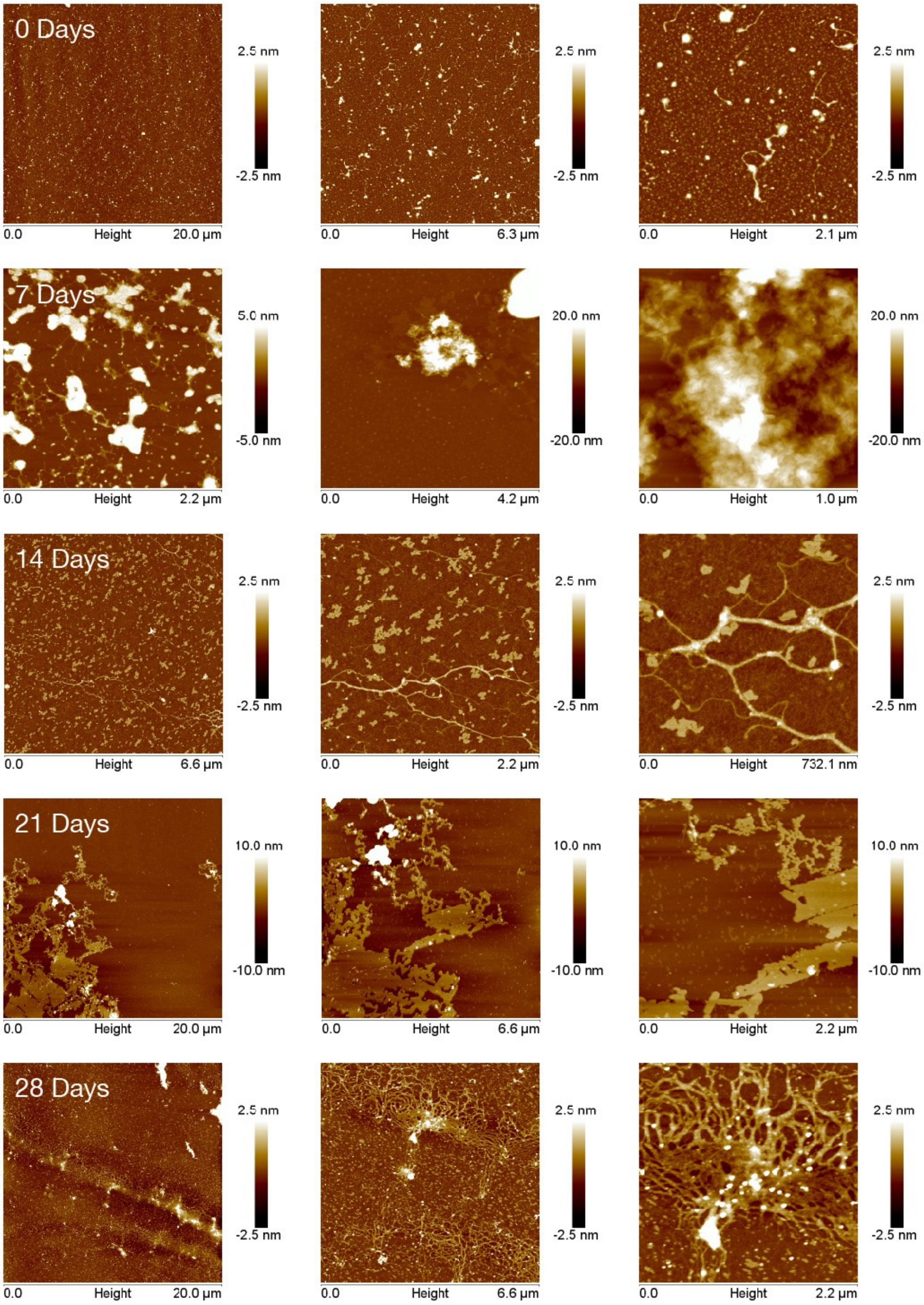

Figure S9. AFM images of 1:1 mixture of **ILVAGK** : **A-ILVAGK** in TAMg over 28 days of aging.

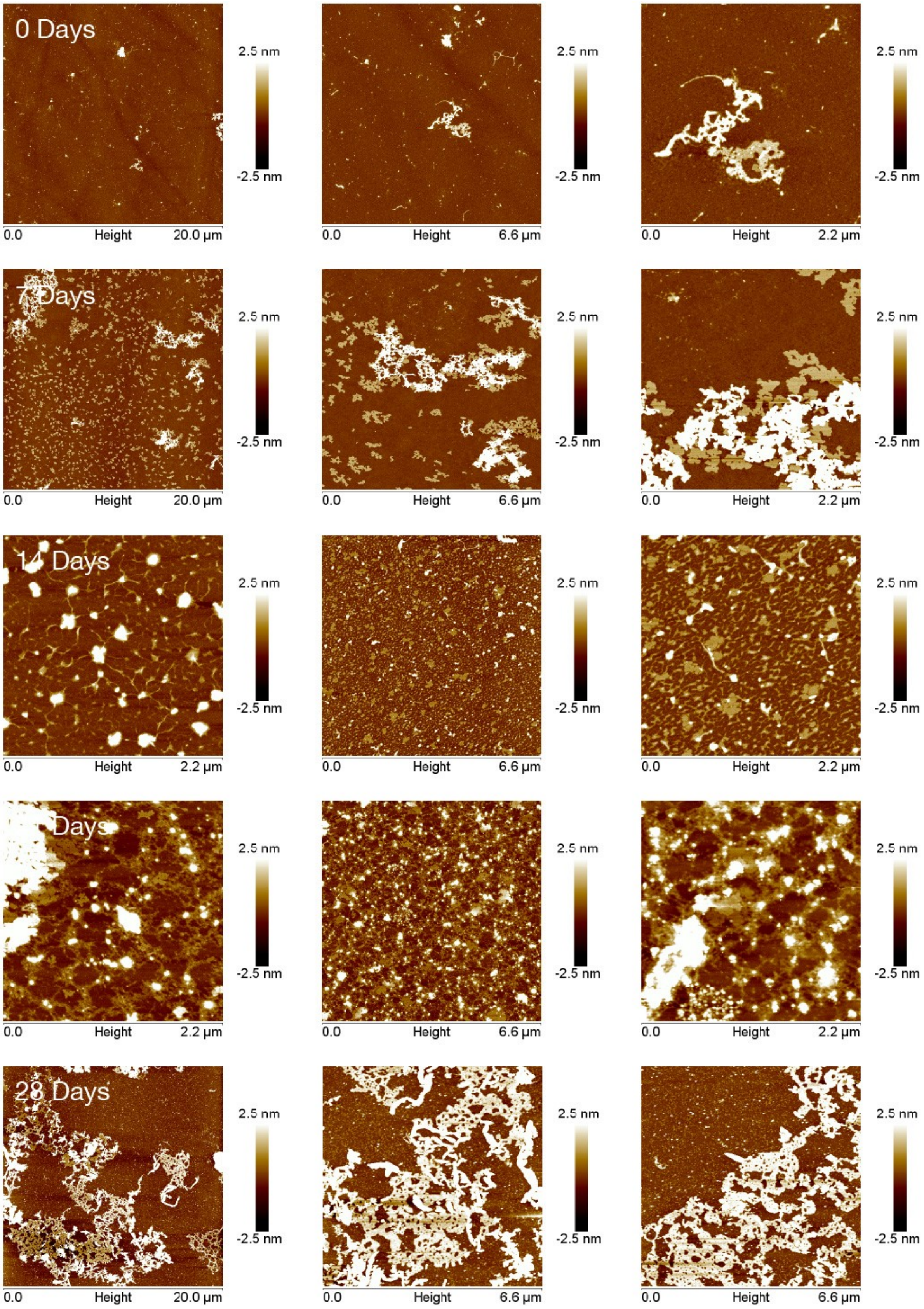

Figure S10. AFM images of 1:0.5 mixture of **ILVAGK** : **A-ILVAGK** in TAMg over 28 days of aging.

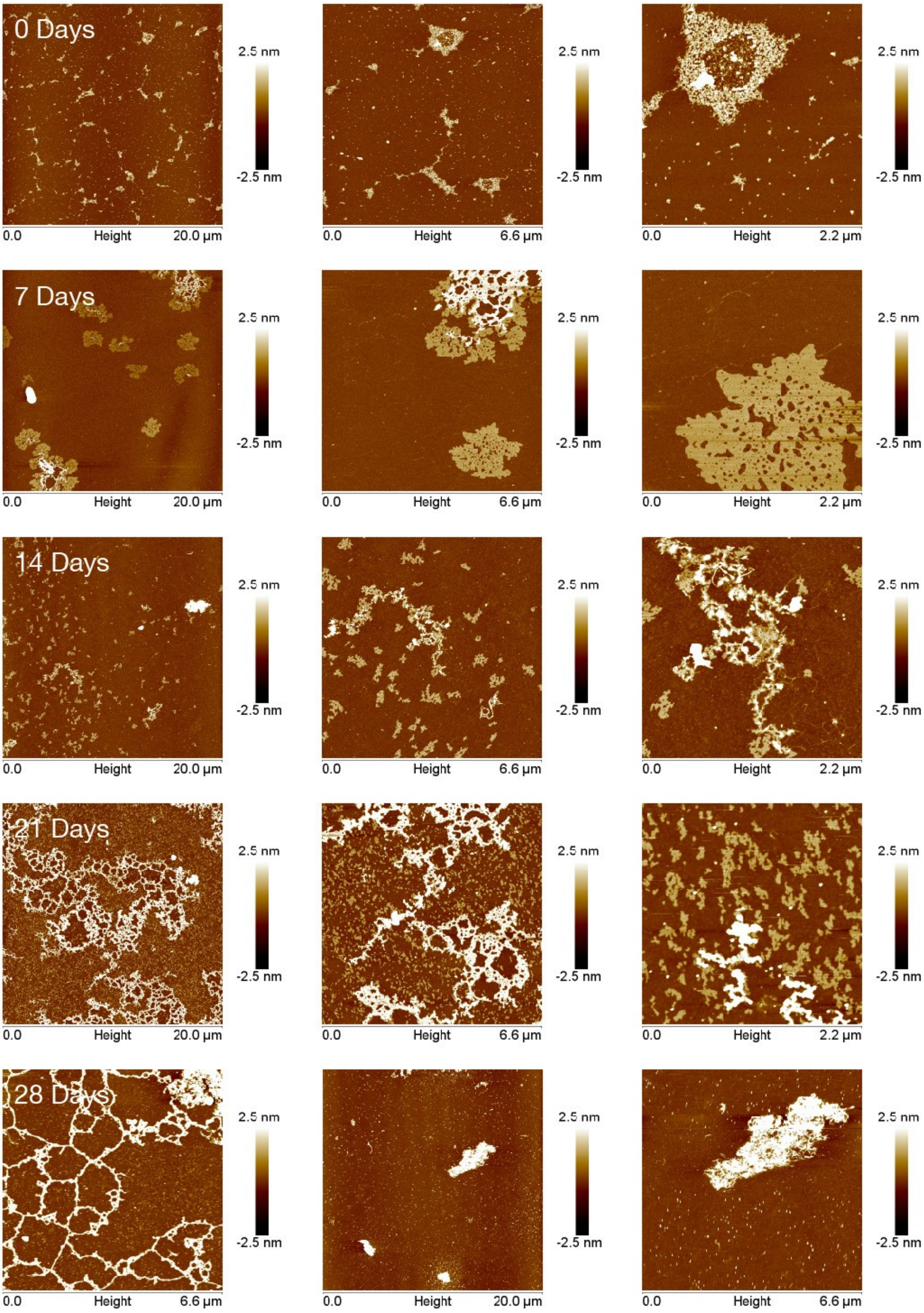

Figure S11. AFM images of 1:0.1 mixture of **ILVAGK** : **A-ILVAGK** in TAMg over 28 days of aging.

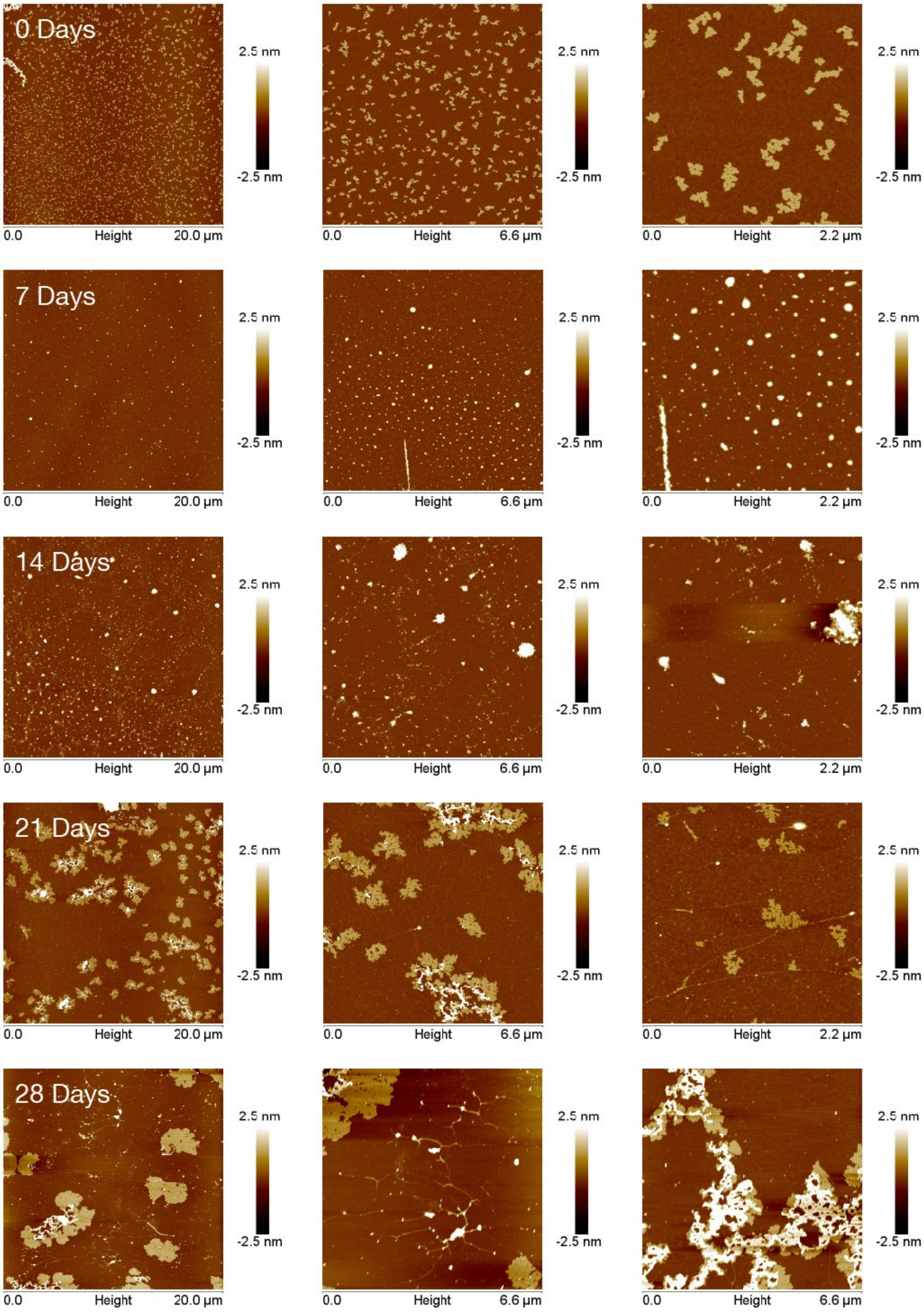

Figure S12. AFM images of 1:0.01 mixture of **ILVAGK** : **A-ILVAGK** in TAMg over 28 days of aging.

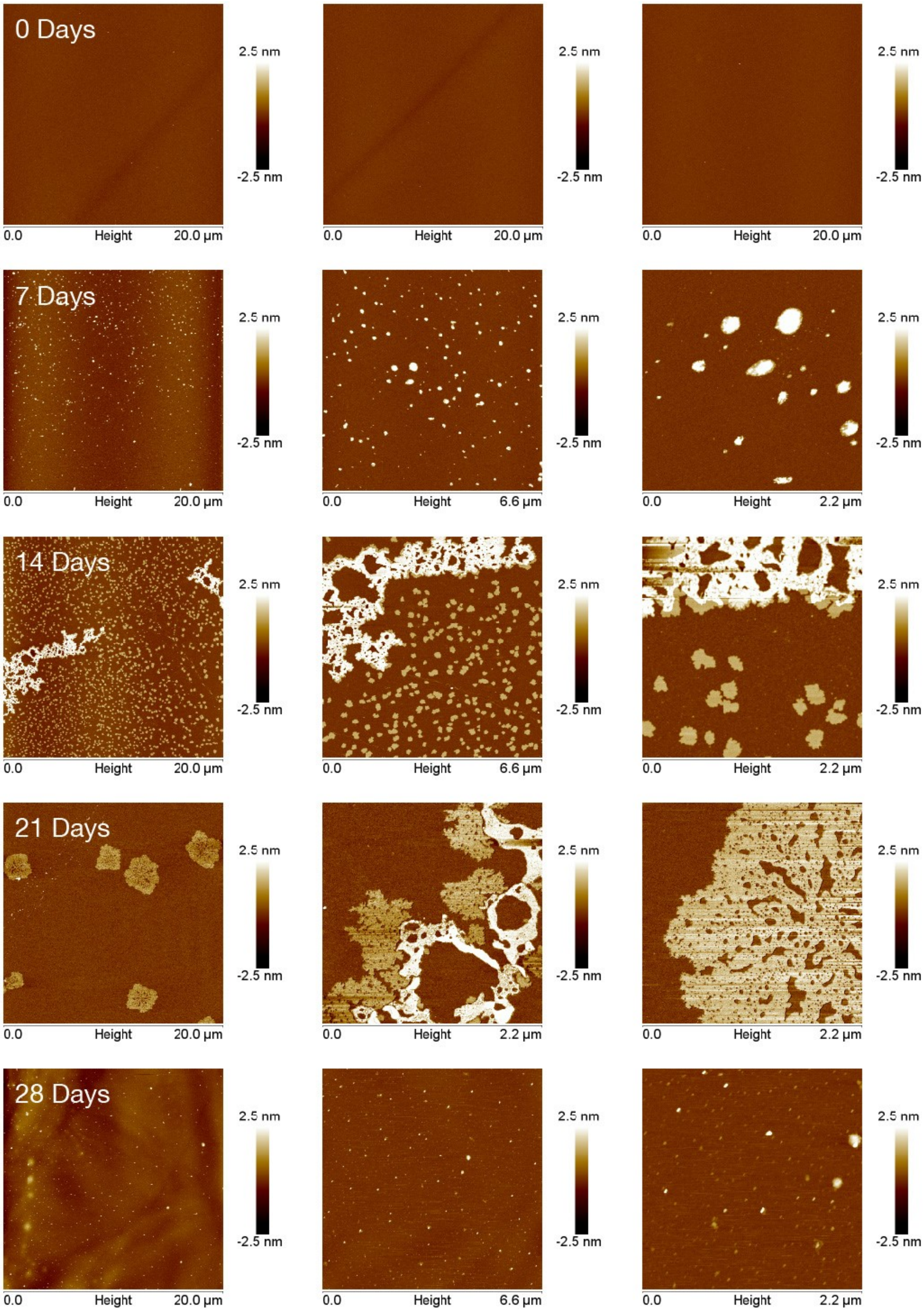

Figure S13. AFM images of **ILVAGK** in 1x TAMg buffer over 28 days of aging (no thermocycling).

Study 2

Sample preparation: Three 500 µL samples of; 20 µM (143.8 µL), 40 µM (287.8 µL) and 60 µM (431.6 µL) were prepared from a 69.5 µM stock of **A-ILVAGK** in TAMg buffer. A 500 µL, 20 µM sample of **A'** was prepared from a 1 mM (10 µL) stock in TAMg buffer and added to the 20 µM sample of **A-ILVAGK**. A 500 µL, 20 µM sample of **2A'** was prepared from a 666.7 µM (15 µL) stock in TAMg buffer and added to the 40 µM sample of **A-ILVAGK**. A 500 µL, 20 µM sample of **3A'** was prepared from a 625 µM (16 µL) stock in TAMg buffer and added to the 60 µM sample of **A-ILVAGK**. The samples were heat-cooled in a thermocycler by the following program: 55 °C for 10 mins, 35 °C for 20 mins, cool to 4 °C. Samples were analysed by TAMg PAGE to assess successful hybridisation of the DNA double helix. Samples were stored at room temperature; in the dark and in 200 µL aliquots. Aliquots were extracted at 0, 7, 14, 21 and 28 days of aging for AFM, aliquots were further analysed by DLS and CD.

Control sample preparation: A 500 µL 20 µM solution of **A-T5** was prepared from a 100 µM stock by diluting 100 µL of **A-T5** with 400 µL of TAMg buffer. A 500 µL 20 µM solution of **A'-T5** was prepared from a 100 µM stock by diluting 100 µL of **A'-T5** with 400 µL of TAMg buffer solution. The two were combined and heat-cooled in a thermocycler by the following program: 55 °C for 10 mins, 35 °C for 20 mins, cool to 4 °C. Two 1000 µL, 10 µM samples were prepared from 100 µM stocks by diluting 50 µL with 950 µL of TAMg buffer. A 10 µM sample of **ILVAGK** was prepared from a 1 mM stock by diluting 10 µL with 990 µL of TAMg buffer. Samples were stored at room temperature; in the dark and in 200 µL aliquots. Aliquots were extracted at 0, 7, 14, 21 and 28 days of aging for AFM, aliquots were further analysed by DLS and CD.

DLS:

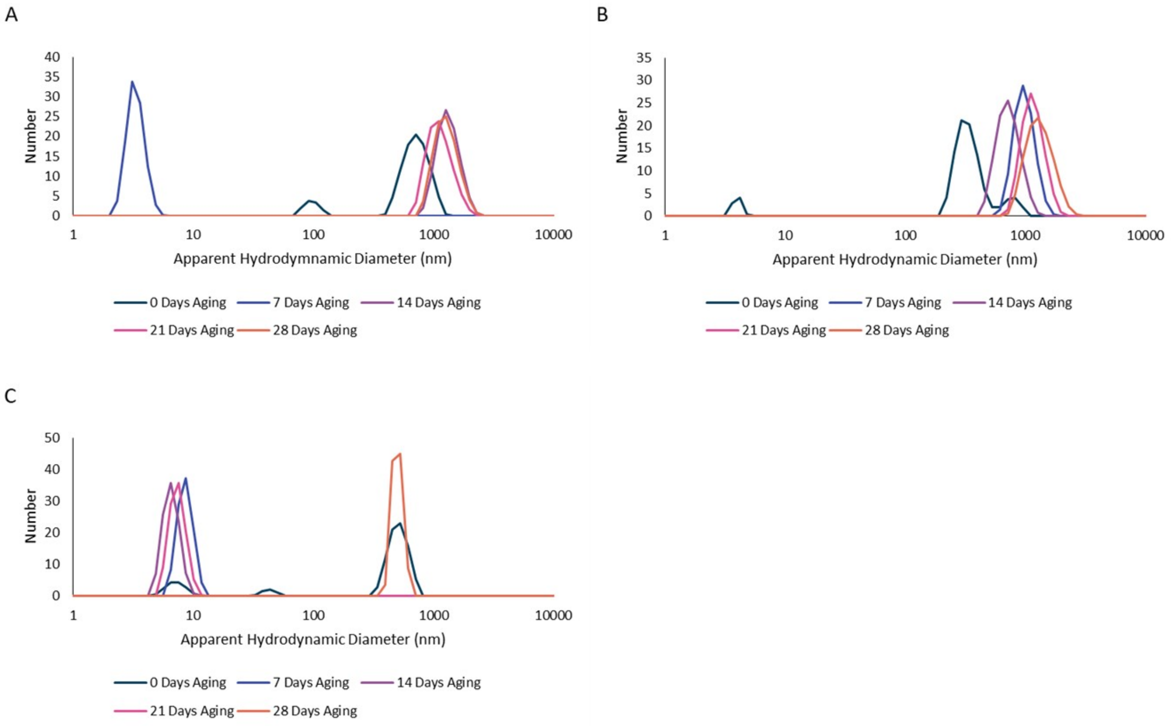

Figure S14. DLS data for Study 2. A: **A-ILVAGK** + **A'**, B: **A-ILVAGK** + **2A'**, and **C: A-ILVAGK + 3A'**. All measured in TAMg buffer and at a concentration of 10 µM.

CD:

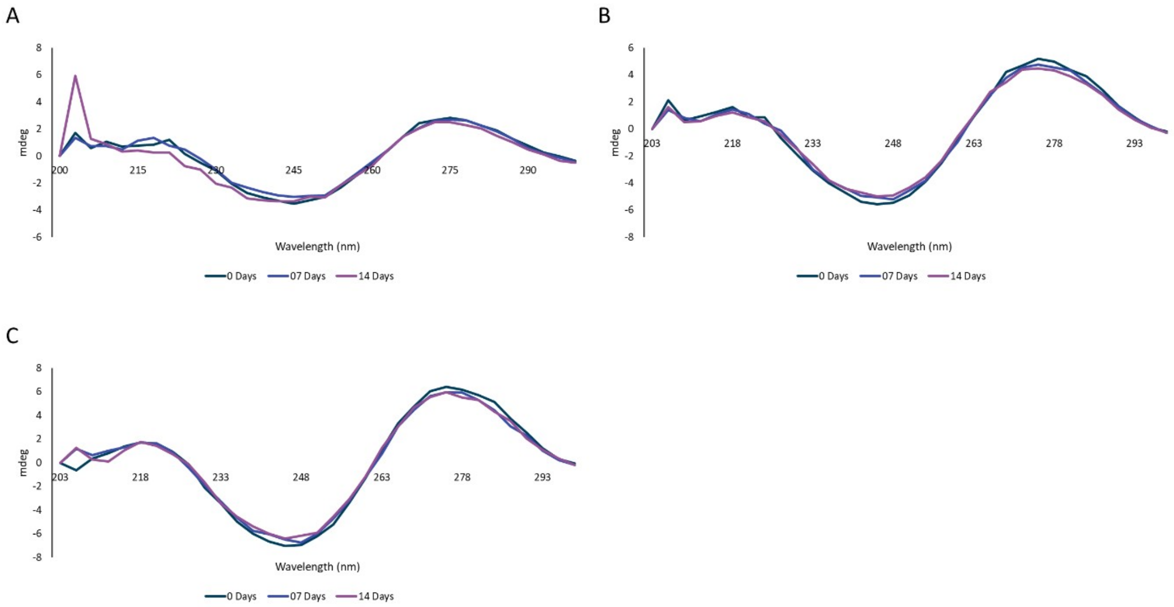

Figure S15. CD spectra for Study 1, A: **A-ILVAGK** + **A'**; B: **A-ILVAGK** + **2A'**; and C: **A-ILVAGK** + **3A'** All measured in TAMg buffer and at a concentration of 10 μM.

AFM:

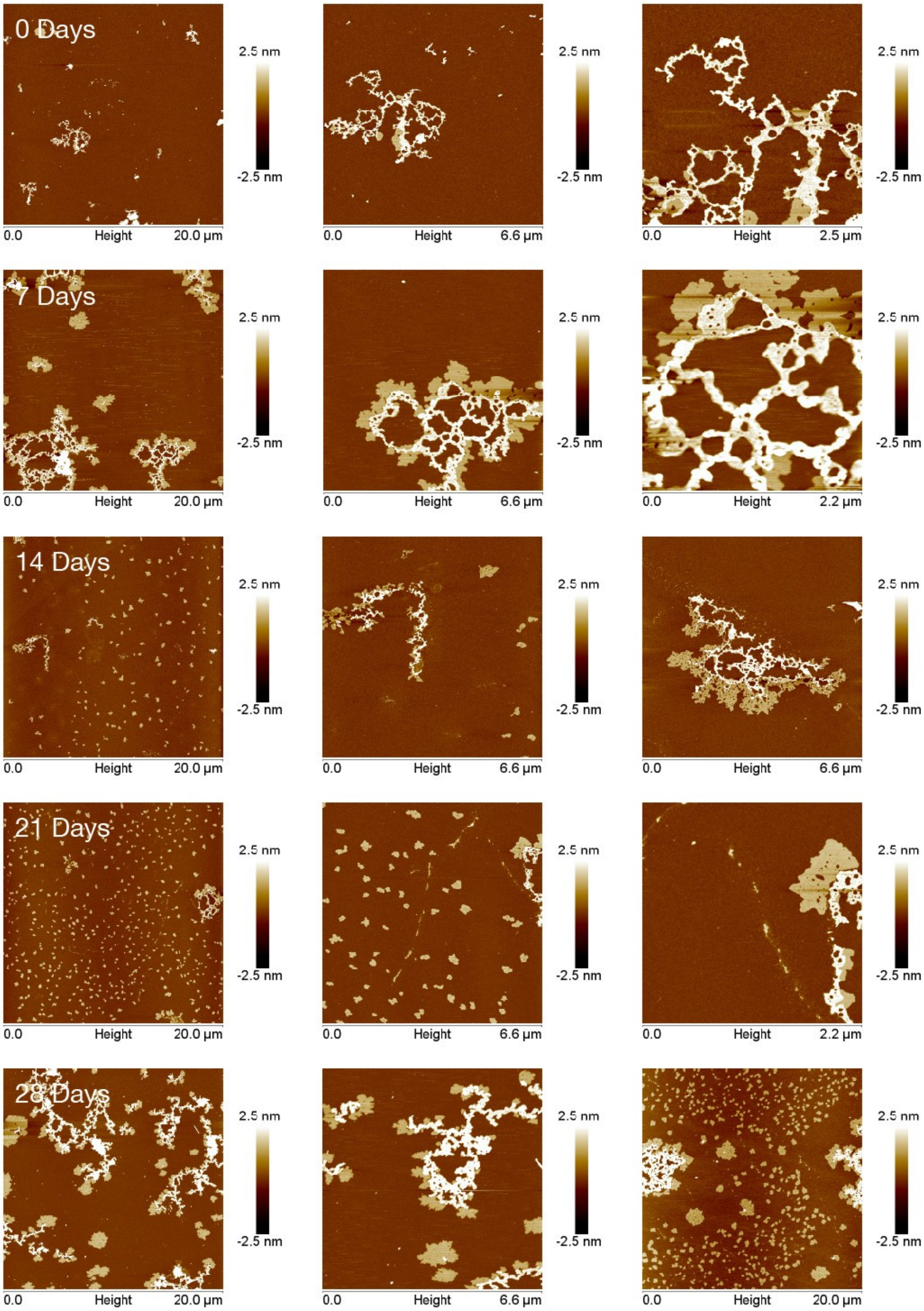

Figure S16. AFM images of **A-ILVAGK** + **A'** over 28 days of aging.

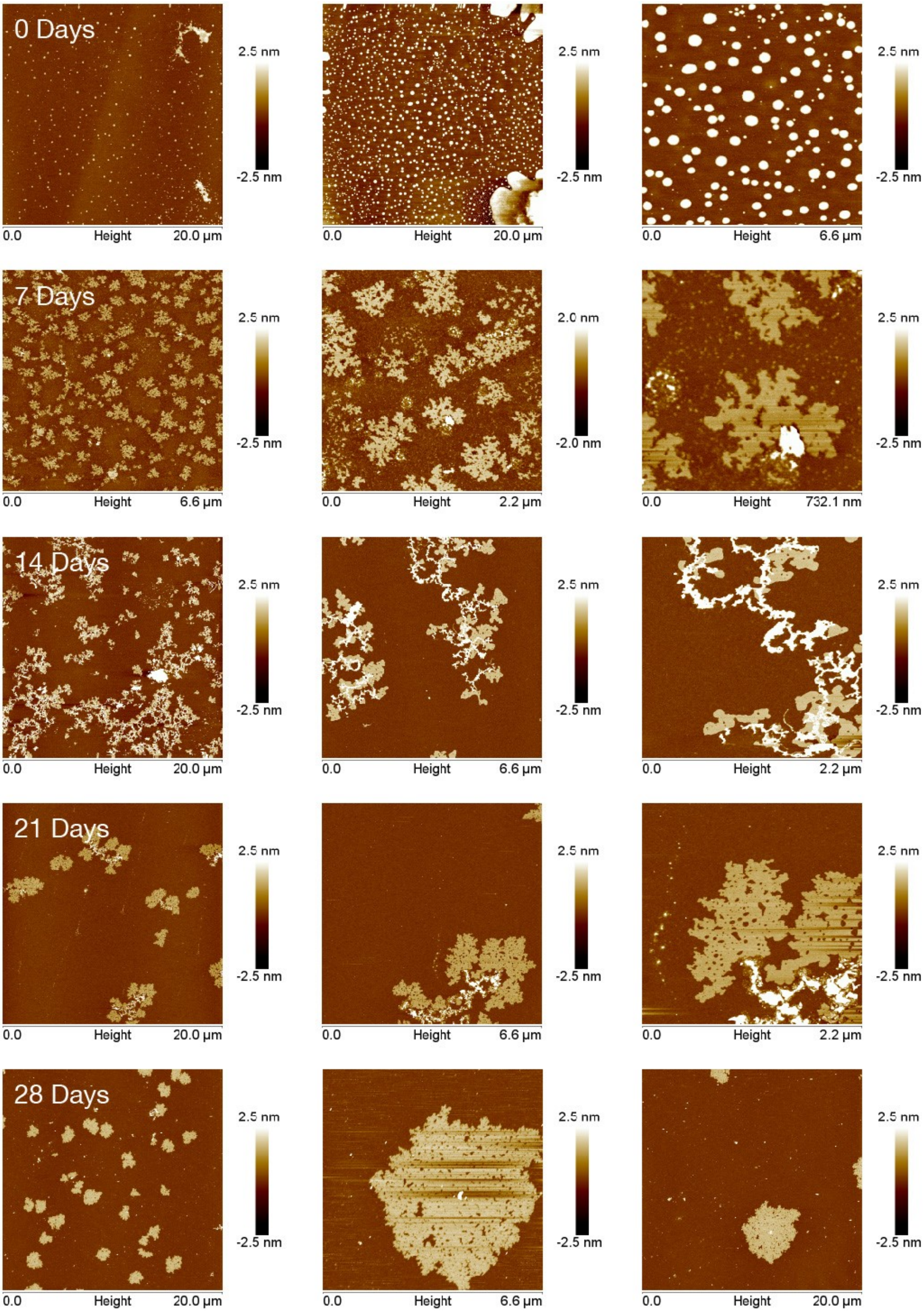

Figure S17. AFM images of sample **A-ILVAGK** + **2A’** over 28 days of aging.

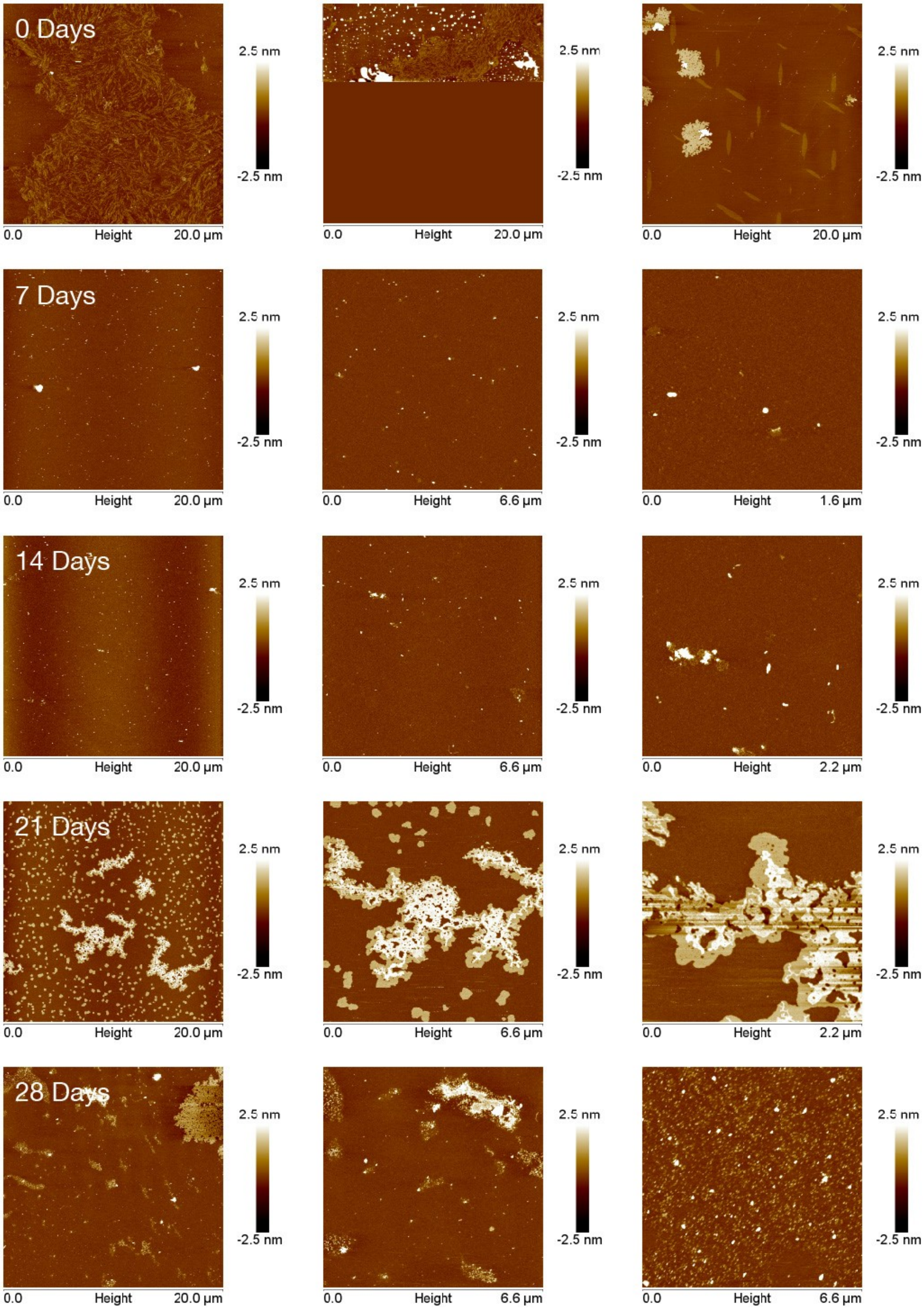

Figure S18. AFM images of **A-ILVAGK** + **3A'** over 28 days of aging.

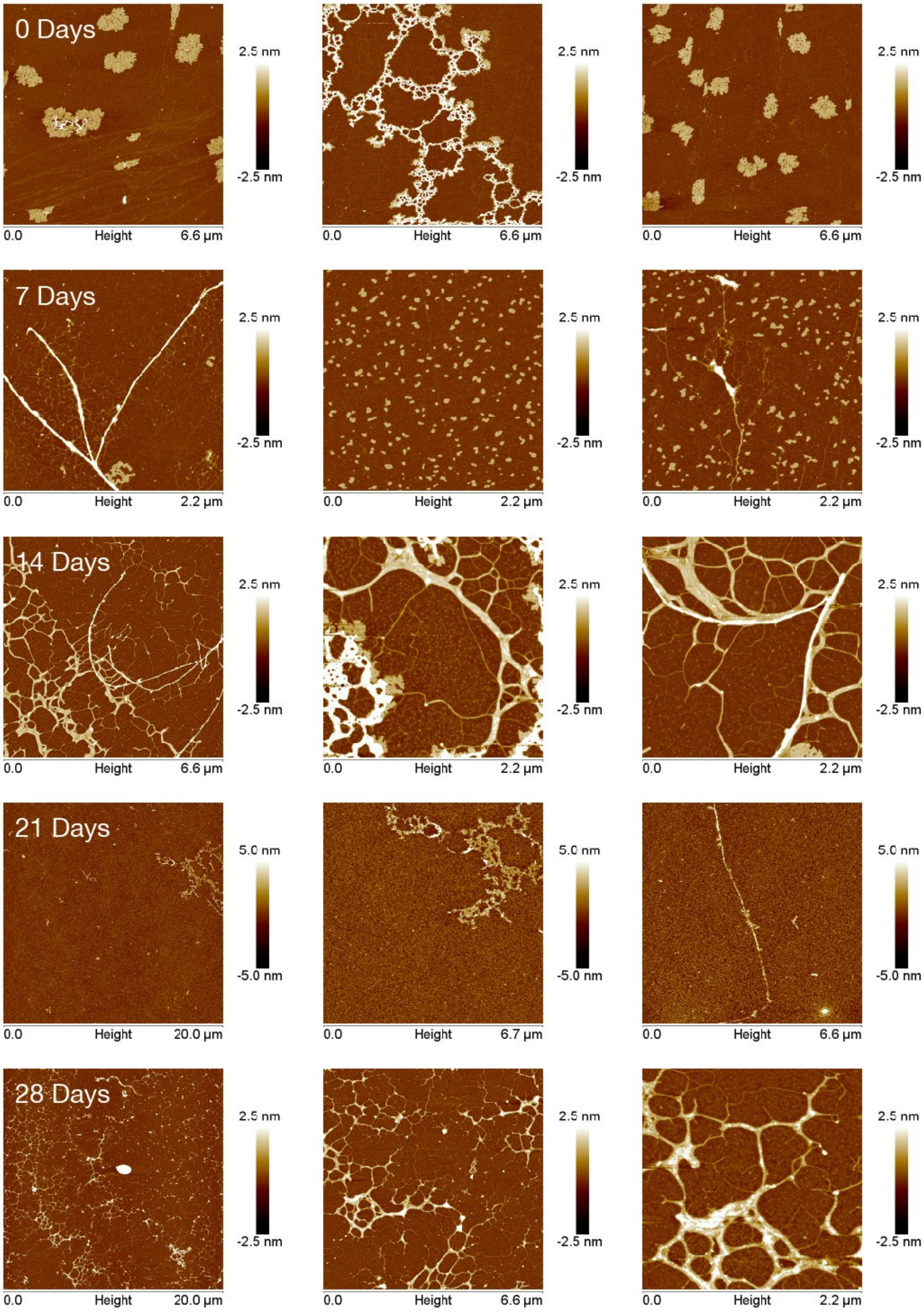

Figure S19. AFM images of 1:1 mixture of DNA **A** and **IVLAGK** in 1x TAMg buffer over 28 days of aging after thermocycling.

Study 3

Sample preparation: A 500 µL, 20 µM solution of **A'-ILVAGK** was prepared from a 42.5 µM stock by diluting 235.3 µL of **A'-ILVAGK** with 264.7 µL of 1 % SDS in filtered autoclaved water. A 500 µL, 20 µM sample of A was prepared from a 100 µM (100 µL) stock in 1 % SDS buffer and added to the 20 µM sample of **A'-ILVAGK**. The sample was heat-cooled in a thermocycler by the following program: 55 °C for 10 mins, 35 °C for 20 mins, cool to 4 °C. A sample of 10 µM **ILVAGK** in 1000 µL of filtered 1 % SDS buffer was prepared by diluting a 1 mM (10 µL) stock of ILVAGK with 990 µL of filtered 1 % SDS buffer. As samples were prepared from stocks in 1 % SDS solution final SDS concentration varies between 0.95 and 0.64 %. Samples were analysed by TAMg PAGE to assess successful hybridisation of the DNA double helix (Fig.4c in paper). Samples were stored at room temperature; in the dark and in 200 µL aliquots. Aliquots were extracted at 0, 7, 14, 21 and 28 days of aging for AFM, aliquots were further analysed by DLS and CD.

Control sample preparation: A 500 µL 20 µM solution of **A-T5** was prepared from a 100 µM stock by diluting 100 µL of **A-T5** with 400 µL of 1 % SDS buffer. A 500 µL 20 µM solution of **A'-T5** was prepared from a 100 µM stock by diluting 100 µL of **A'-T5** with 400 µL of 1 % SDS solution. The two were combined and heat-cooled in a thermocycler by the following program: 55 °C for 10 mins, 35 °C for 20 mins, cool to 4 °C. Two 1000 µL,10 µM samples were prepared from 100 µM stocks by diluting 50 µL with 950 µL of 1 % SDS buffer. Samples were stored at room temperature; in the dark and in 200 µL aliquots. Aliquots were extracted at 0, 7, 14, 21 and 28 days of aging for AFM, aliquots were further analysed by DLS and CD.

DLS:

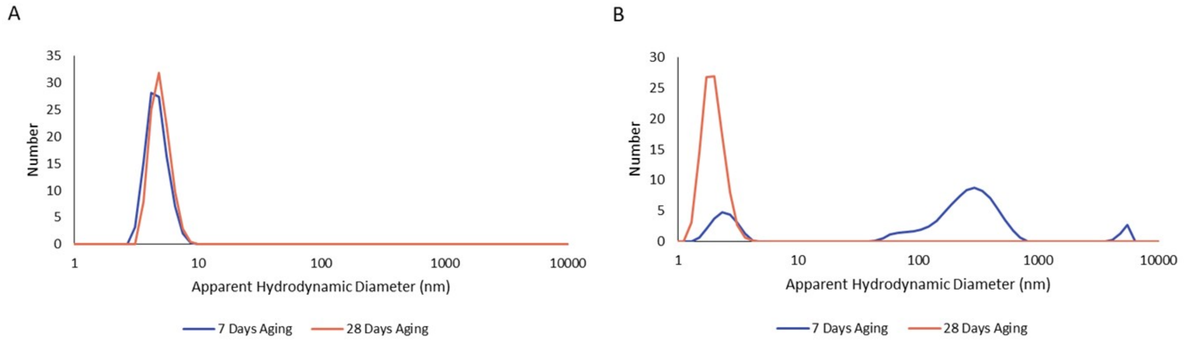

Figure S20. DLS data for study 3. A: **A'-ILVAGK** + A, B: **ILVAGK**. All measured in 1 % SDS buffer and at a concentration of 10 µM.

CD:

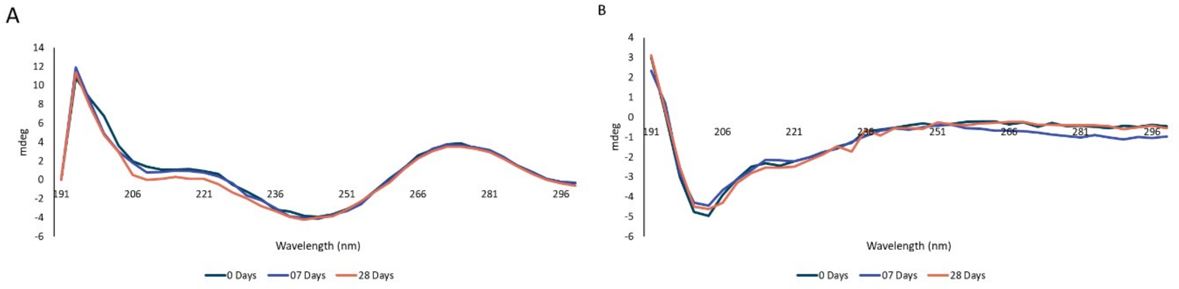

Figure S21. CD spectra for A: **A'-ILVAGK** + **A**, B: **ILVAGK**. All measured in 1 % SDS buffer and at a concentration of 10 µM.

AFM:

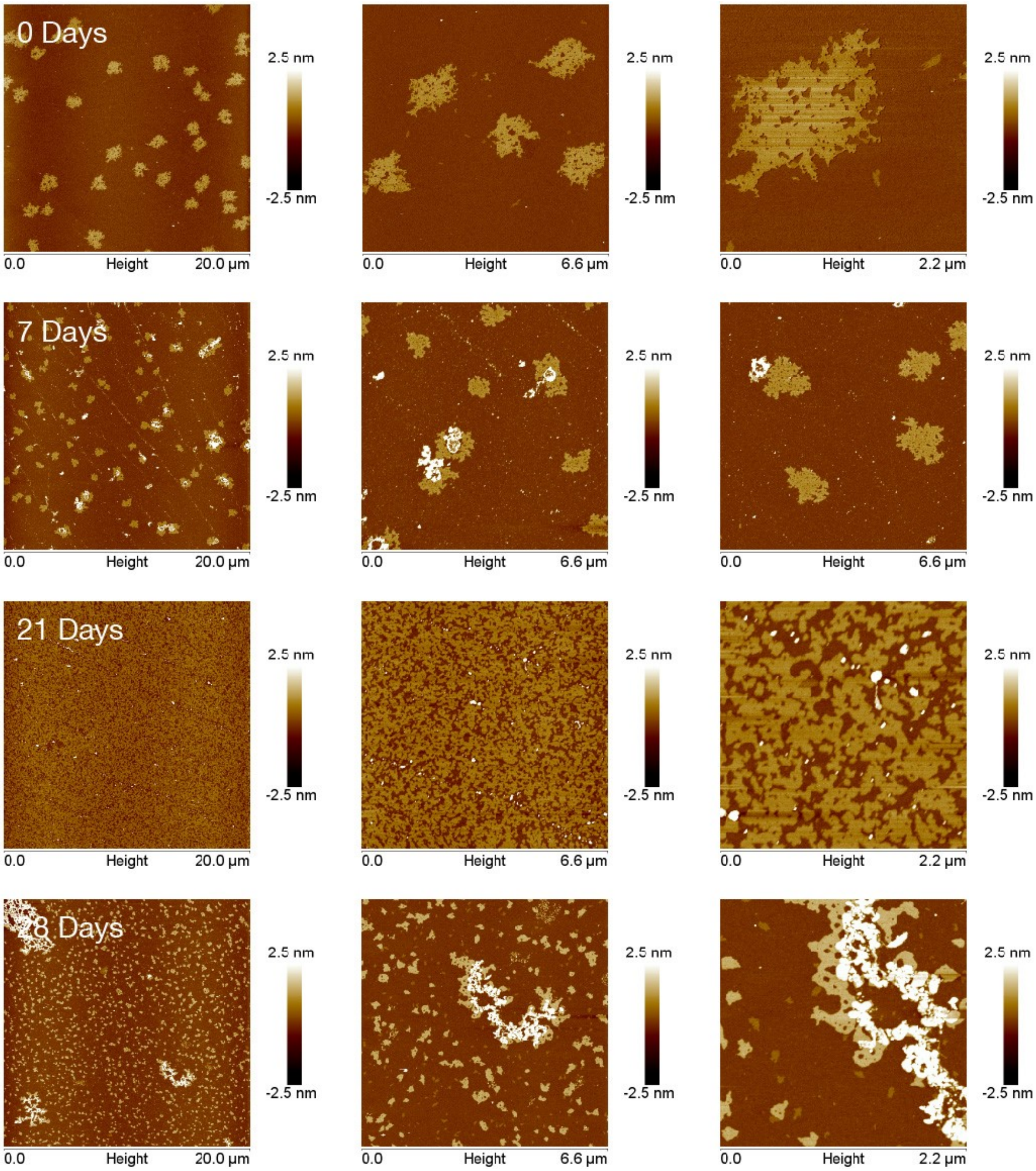

Figure S22. AFM images of sample **A'-ILVAGK** + **A** over 28 days of aging, deposited from 1% SDS.

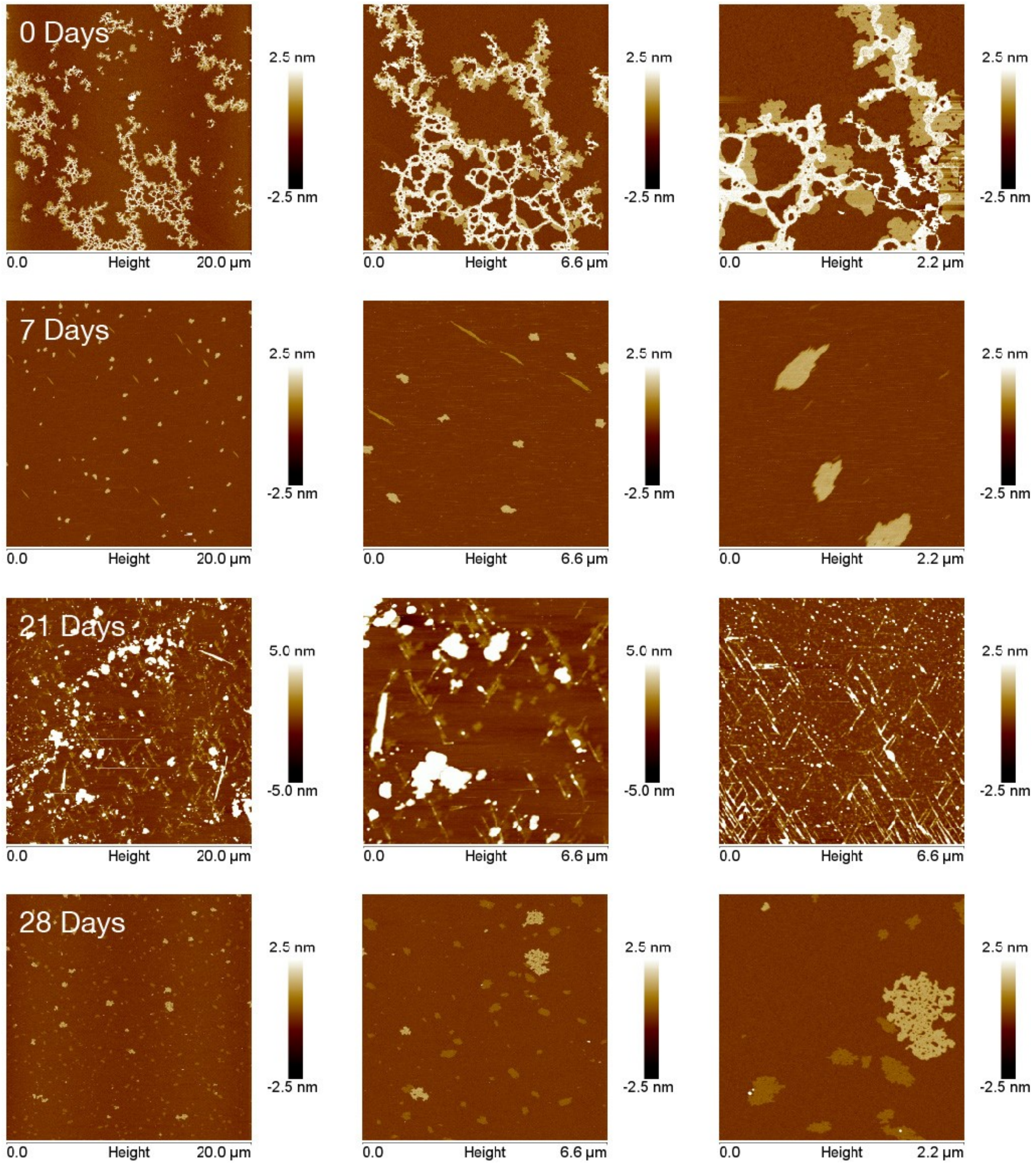

Figure S23. AFM images of **ILVAGK** over 28 days of aging, deposited from 1% SDS.

Control samples

DLS:

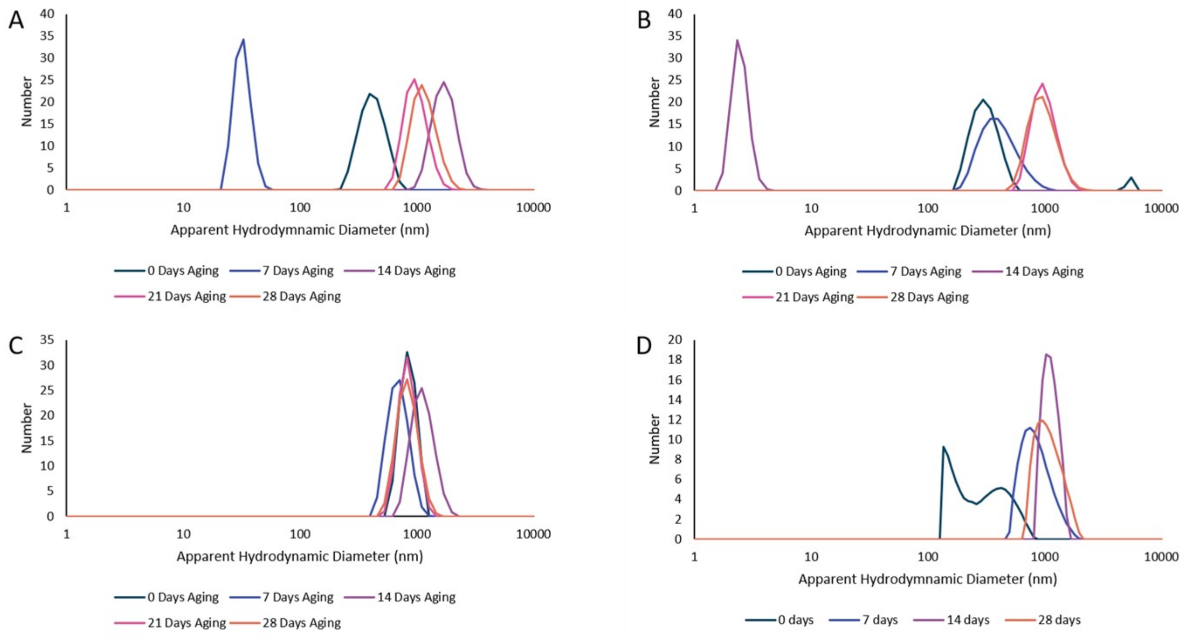

Figure S24. DLS control data for studies 1 and 2. A: **A** (low scattering intensity), B: **A'** (low scattering intensity), C: **ILVAGK**, and D: **A** + **ILVAGK** (unconjugated). All samples at a concentration of 10 µM and in TAMg buffer.

CD:

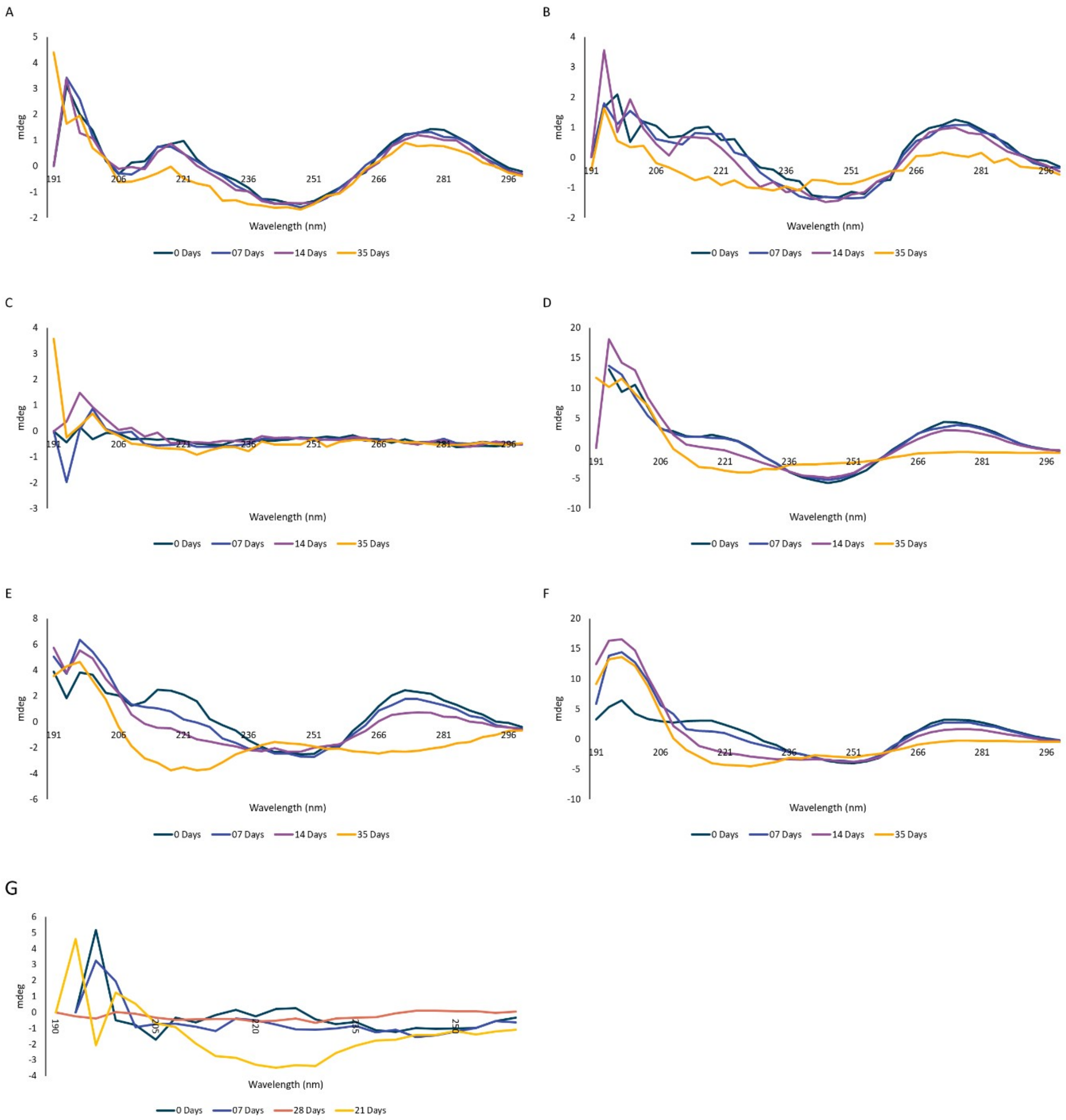

Figure S25. CD spectra of controls for Studies 1 and 2. A: **A**, B: **A’**, C: **ILVAGK**, D: **A** + **A’** hybridised, E: **2A’**, F: **3A’**, and G: 1:1 mixture of **A** and **ILVAGK**. All samples at a concentration of 10 µM and in TAMg buffer.

AFM:

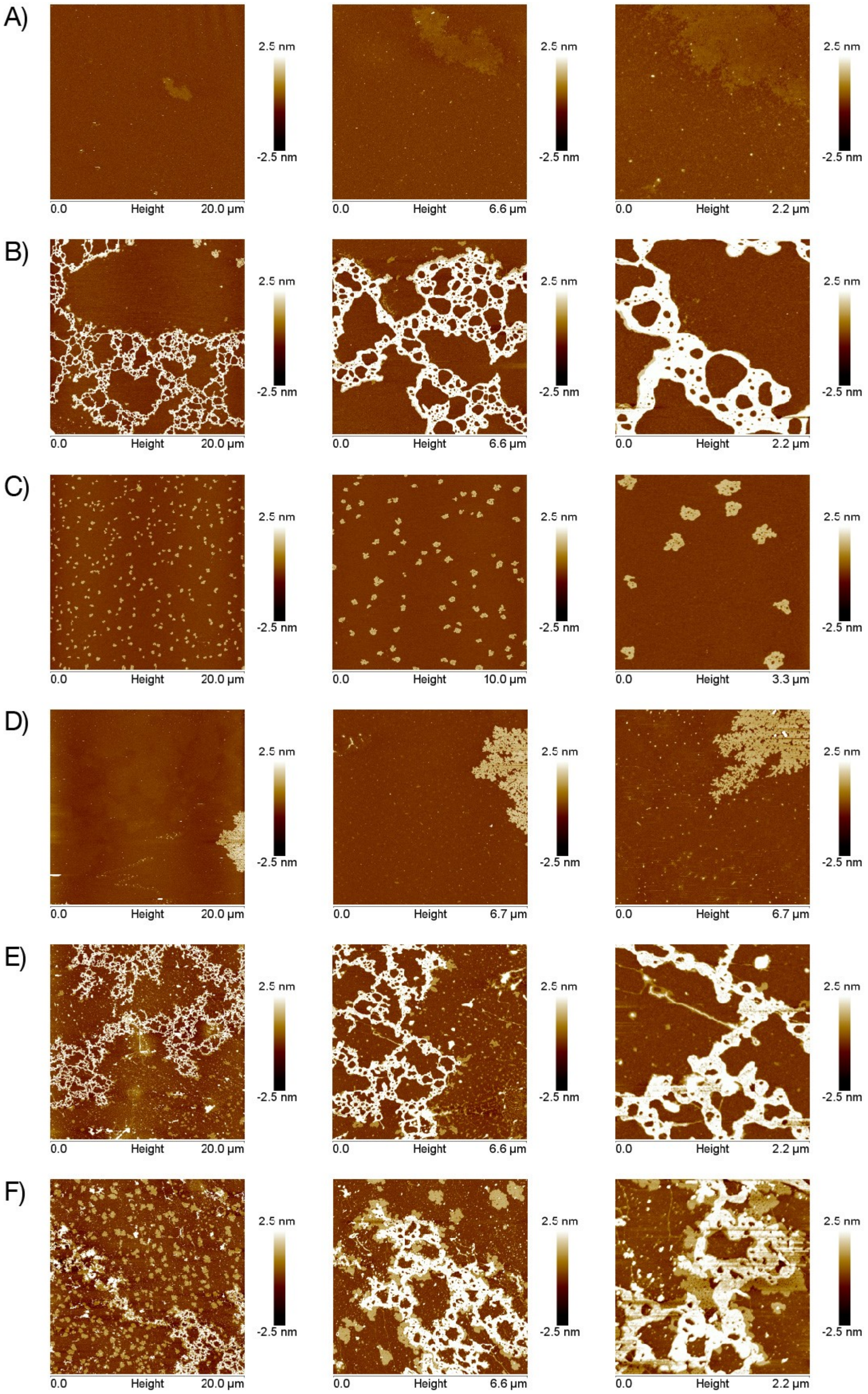

Figure S26. AFM images of DNA controls in 1x TAMg buffer over 28 days of aging. A: 1x TAMg, B: **A**, C: **A'**, D: **A+A'**, E: **2A'** and F: **3A'**.

DLS:

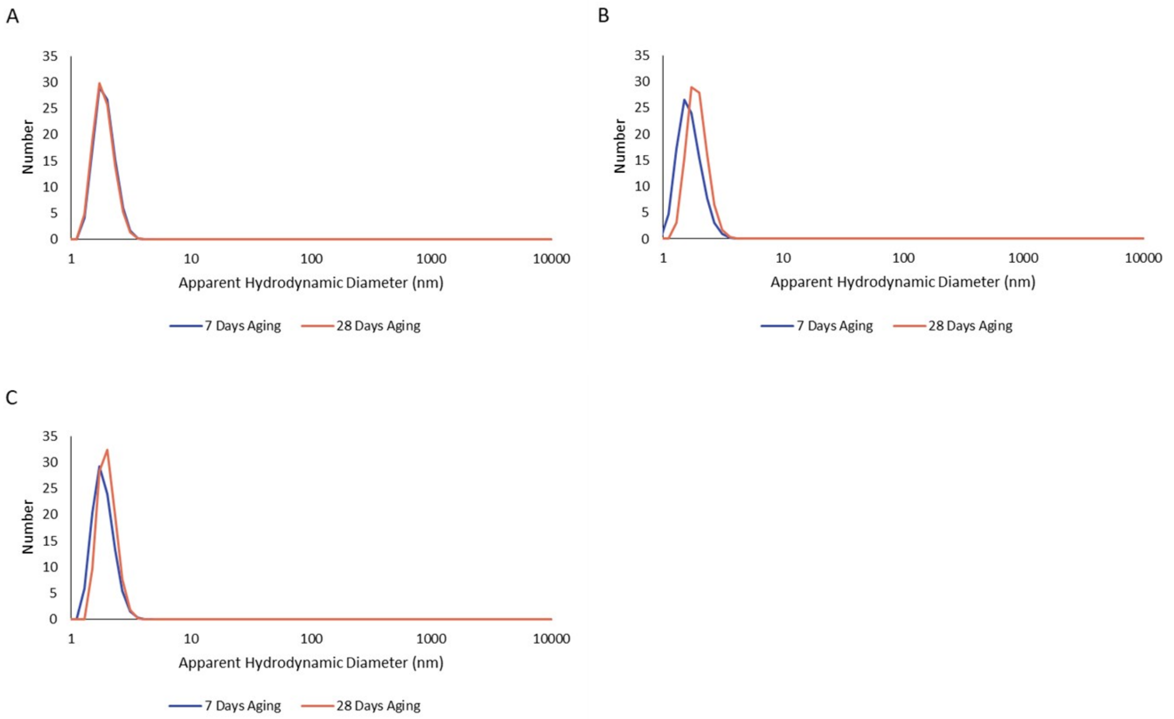

Figure S27. DLS control data for Study 3. A: **A**, B: **A’**, C: **A + A’** hybridised. All samples at a concentration of 10 µM and in 1 % SDS buffer.

CD:

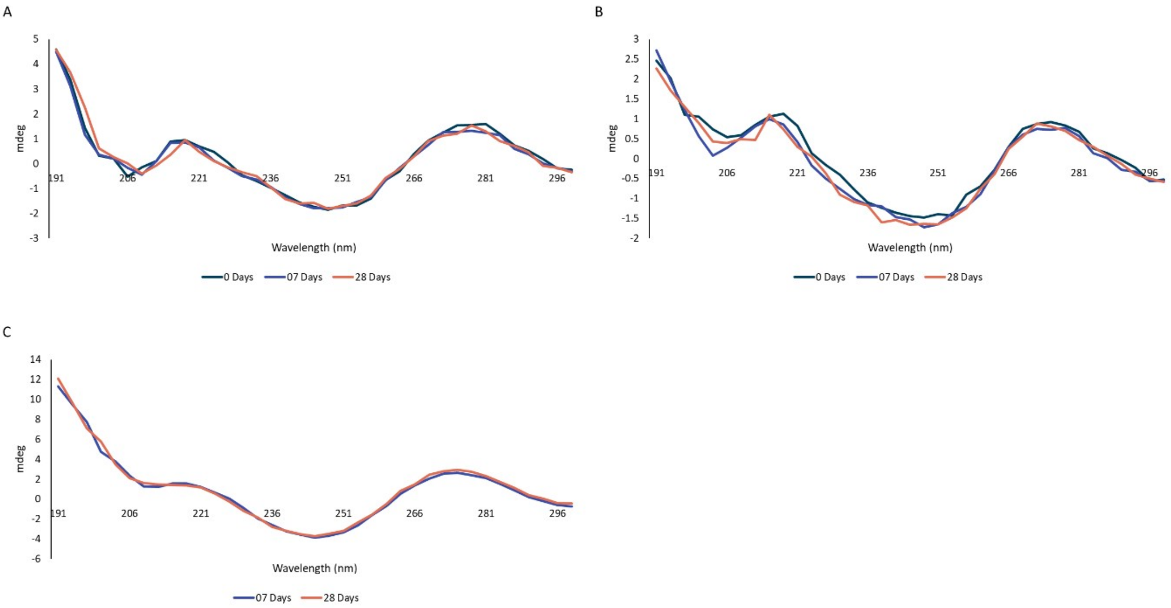

Figure S28. CD spectra of controls for Study 3. A: **A**, B: **A’**, and C: **A + A’** hybridised. All samples at a concentration of 10 µM and in 1 % SDS buffer.

AFM:

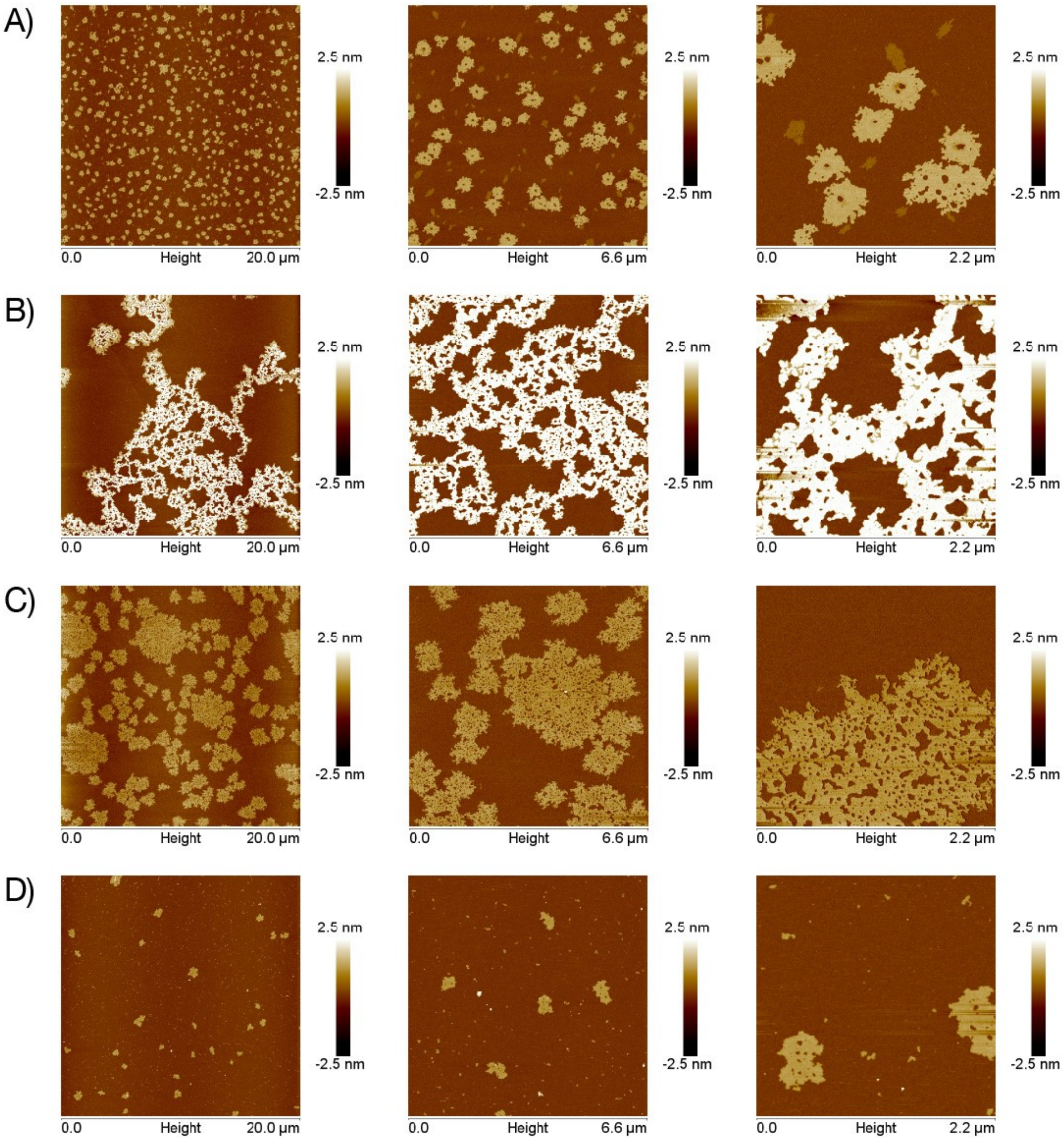

Figure S29. AFM images of controls in 1 % SDS buffer after 28 days of aging. **A**: 1 % SDS, B: **A**, C: **A'**, D: **A+A'**.
